# Supplementary material for: Preventing an Antigenically Disruptive Mutation in Egg-Based H3N2 Seasonal Influenza Vaccines by Mutational Incompatibility
Source: Cell Host Microbe. 2019 Jun 12;25(6):836–844.e5. doi: 10.1016/j.chom.2019.04.013 (PMC6579542; doi:10.1016/j.chom.2019.04.013)
Supplement: Document S1. Figures S1–S4 and Tables S1–S3 [file mmc1.pdf]

Cell Host & Microbe, Volume *25*

Supplemental Information

Preventing an Antigenically Disruptive Mutation in Egg-Based H3N2 Seasonal Influenza Vaccines by Mutational Incompatibility

Nicholas C. Wu, Huibin Lv, Andrew J. Thompson, Douglas C. Wu, Wilson W.S. Ng, Rameshwar U. Kadam, Chih-Wei Lin, Corwin M. Nycholat, Ryan McBride, Weiwen Liang, James C. Paulson, Chris K.P. Mok, and Ian A. Wilson

Table S1. Number of HA sequences in the egg-adaptive mutation analysis, Related to Figure 1.

Year	No passaged	Egg-passaged
**2003**	33	10
**2004**	9	8
**2005**	41	3
**2006**	18	6
**2007**	78	26
**2008**	37	17
**2009**	269	33
**2010**	138	33
**2011**	227	34
**2012**	348	68
**2013**	425	47
**2014**	713	64
**2015**	1,490	56
**2016**	1,993	15
**2017**	4,985	57
**2018**	2,334	52

Table S2. H3N2 components of influenza vaccines, Related to Figure 1.

Season	Strain name	Accession	Reference
**2006-2007**	A/Wisconsin/67/2005 (NYMC X-161)	GenBank: ACF41911.1	https://www.ema.europa.eu/documents/assessment-report/intanza-epar-public-assessment-report_en.pdf
**2009-2010**	A/Uruguay/716/2007 (NYMC X-175C)	GenBank: AIE52751.1	http://www.who.int/immunization_standards/vaccine_quality/fluzone_sanofi_pasteur_product_insert.pdf
**2010-2011**	A/Victoria/210/2009 (NYMC X-187)	GenBank: ADO29878.1	https://ec.europa.eu/health/documents/community-register/2010/2010081886928/anx_86928_en.pdf
**2011-2012**			https://ec.europa.eu/health/documents/community-register/2012/20120206116749/anx_116749_en.pdf
**2012-2013**	A/Victoria/361/2011 (IVR-165)	GISAID: EPI551807	http://ec.europa.eu/health/documents/community-register/2012/20120822124238/anx_124238_en.pdf
**2013-2014**	A/Texas/50/2012 (NYMC X-223A)	GenBank: AIE52715.1	https://ec.europa.eu/health/documents/community-register/2013/20130924126650/anx_126650_en.pdf
**2014-2015**			https://ec.europa.eu/health/documents/community-register/2014/20140806129489/anx_129489_en.pdf
**2015-2016**	A/South Australia/55/2014 (IVR-175)	GISAID: EPI696967	https://ec.europa.eu/health/documents/community-register/2015/20150825132942/anx_132942_en.pdf
**2016-2017**	A/Hong Kong/4801/2014 (NYMC X-263B)	GISAID: EPI614414	https://ec.europa.eu/health/documents/community-register/2016/20161103136374/anx_136374_en.pdf
**2017-2018**			https://www.ema.europa.eu/documents/product-information/intanza-epar-product-information_en.pdf
**2018**	A/Singapore/INFIMH-16-0019/2016 (NIB-104)	GISAID: EPI1082230	http://www.medsafe.govt.nz/profs/Datasheet/f/fluquadriinj.pdf
**2018-2019**	A/Singapore/INFIMH-16-0019/2016 (IVR-186)	GISAID: EPI1151840	https://www.vaccineshoppecanada.com/document.cfm?file=fluzone_qiv_e_2018.pdf
**2019**	A/Switzerland/8060/2017 (NIB-112)	GISAID: EPI_ISL_331787	http://origin.who.int/influenza/vaccines/virus/candidates_reagents/summary_a_h3n2_cvv-egg_sh19.pdf

Table S3. X-ray data collection and refinement statistics, Related to Figure 3, Figure 4 and Figure 5.

**Data collection**	IVR-165 Apo	IVR-165 + 3'SLNLN	IVR-165 + 6'SLNLN	Bris07 G186V/L194P Apo	Bris07 G186V/L194 + 3'SLNLN	Bris07 G186V/L194 + 6'SLNLN
Beamline	APS 23ID-B	ALS 5.0.3	APS 23ID-B	SSRL 12-2	SSRL 12-2	SSRL 12-2
Wavelength (Å)	1.0332	0.9765	1.0332	0.9795	0.9795	0.9795
Space group	H32	H32	H32	H32	H32	H32
Unit cell parameters (Å and °)	a=b=100.4, c=382.1	a=b=101.5, c=388.1	a=b=101.1, c=387.2	a=b=100.6, c=384.5	a=b=100.2, c=382.8	a=b=100.3, c=383.6
Resolution (Å)	50.00-1.95 (2.01-1.95)[a]	50.00-1.95 (2.02-1.95)[a]	50.00-1.75 (1.81-1.75)[a]	50.00-2.25 (2.33-2.25)[a]	50.00-2.10 (2.18-2.10)[a]	50.00-2.40 (2.49-2.40)[a]
Unique Reflections	54,947 (4,957)[a]	56,657 (5,556)[a]	76,888 (6,920)[a]	36,162 (3,556)[a]	44,480 (4,871)[a]	29,754 (2,921)[a]
Redundancy	18.1 (17.4)[a]	7.5 (7.5)[a]	37.7 (35.5)[a]	19.0 (17.1)[a]	19.2 (19.9)[a]	19.4 (19.4)[a]
Completeness (%)	100.0 (100.0)[a]	99.7 (99.4)[a]	100.0 (100.0)[a]	99.9 (99.9)[a]	100.0 (100.0)[a]	100.0 (100.0)[a]
$\langle I/\sigma_I \rangle$	26.0 (2.8)[a]	33.5 (3.8)[a]	39.0 (1.8)[a]	29.7 (2.9)[a]	20.6 (2.7)[a]	25.5 (2.7)[a]
R_{sym}[b]	0.15 (0.90)[a]	0.11 (0.99)[a]	0.13 (1.33)[a]	0.10 (0.89)[a]	0.13 (0.95)[a]	0.10 (0.92)[a]
R_{pim}[b]	0.04 (0.22)[a]	0.04 (0.38)[a]	0.02 (0.23)[a]	0.02 (0.22)[a]	0.03 (0.21)[a]	0.02 (0.21)[a]
$CC_{1/2}$[c]	1.00 (0.92)[a]	1.00 (0.86)[a]	1.00 (0.92)[a]	1.00 (0.92)[a]	1.00 (0.89)[a]	1.00 (0.92)[a]
Z_a[d]	1	1	1	1	1	1
**Refinement statistics**						
Resolution (Å)	41.86-1.95	42.85-1.95	39.90-1.75	46.82-2.25	46.26-2.10	43.14-2.40
Reflections (work)	51,993	53,635	73,049	34,376	41,587	28,205
Reflections (test)	2,670	2,840	3,825	1,743	2,185	1,448
R_{cryst}(%)[e] / R_{free}(%)[f]	16.7 / 19.3	17.9 / 20.8	16.5 / 18.7	19.8 / 22.8	18.2 / 21.4	18.7 / 23.1
No. of atoms						
Protein	3,975	3,955	3,981	3,898	3,944	3,897
Water	406	423	518	240	368	172
Glycan	201	259	215	183	208	197
Ligand[g]						
Sialic acid	-	20	20	-	21	21
Non-sialic acid	-	37	26	-	-	-
Average *B*-value (Å²)						
Protein	43	39	35	64	50	69
Water	45	46	45	51	47	53
Glycan	77	76	67	101	88	111
Ligand[g]						
Sialic acid	-	66	59	-	104	156
Non-sialic acid	-	109	84	-	-	-
Wilson *B*-value (Å²)	25	28	25	36	29	41
**RMSD from ideal geometry**						
Bond length (Å)	0.010	0.010	0.011	0.011	0.010	0.010
Bond angle (°)	1.49	1.51	1.53	1.46	1.48	1.48
**Ramachandran statistics (%)**						
Favored	96.2	96.2	96.4	95.5	95.4	95.1
Outliers	0.0	0.2	0.0	0.0	0.0	0.0
**PDB code**	6NS9	6NSA	6NSB	6NSC	6NSF	6NSG

[a] Numbers in parentheses refer to the highest resolution shell.

[b] $R_{sym} = \Sigma_{hkl} \Sigma_i | I_{hkl,i} - \langle I_{hkl} \rangle | / \Sigma_{hkl} \Sigma_i I_{hkl,i}$ and $R_{pim} = \Sigma_{hkl} (1/(n-1))^{1/2} \Sigma_i | I_{hkl,i} - \langle I_{hkl} \rangle | / \Sigma_{hkl} \Sigma_i I_{hkl,i}$, where $I_{hkl,i}$ is the scaled intensity of the ith measurement of reflection h, k, l, $\langle I_{hkl} \rangle$ is the average intensity for that reflection, and *n* is the redundancy.

[c] $CC_{1/2}$ = Pearson correlation coefficient between two random half datasets.

[d] Z_a is the number of HA protomers per crystallographic asymmetric unit.

[e] $R_{cryst} = \Sigma_{hkl} | F_o - F_c | / \Sigma_{hkl} | F_o | \times 100$, where F_o and F_c are the observed and calculated structure factors, respectively.

[f] R_{free} was calculated as for R_{cryst}, but on a test set comprising 5% of the data excluded from refinement.

[g] Ligand indicates 3'SLNLN or 6'SLNLN

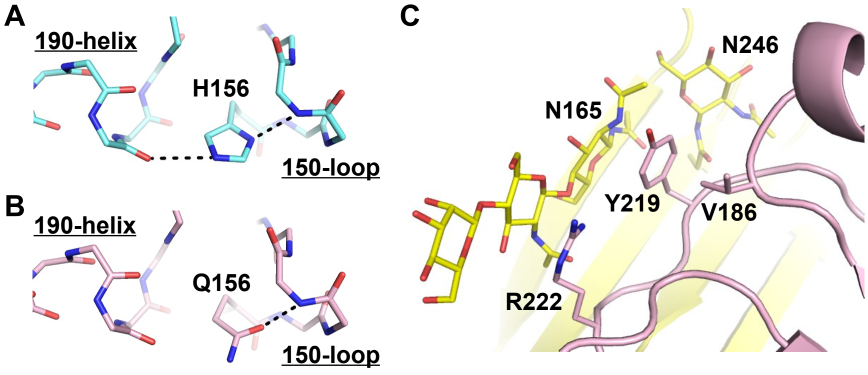

Figure S1. Structural characterization of IVR-165 HA, Related to Figure 3.

(A, B) Hydrogen bond interactions between residue 156 and main chain of the 150-loop (A) in Vic11 HA and in (B) IVR-165 HA are shown. In Vic11 HA, residue 156 also forms a hydrogen bond with the main chain of the 190-helix. Such an interaction is absent in IVR-165 HA.

(C) Stacking interaction between Y219 and the first N-acetyl glucosamine of the N-glycan at residue 165 from the neighboring HA protomer (yellow) is shown. R222 also forms a stacking interaction with the second N-acetyl glucosamine of the same N-glycan at residue 165.

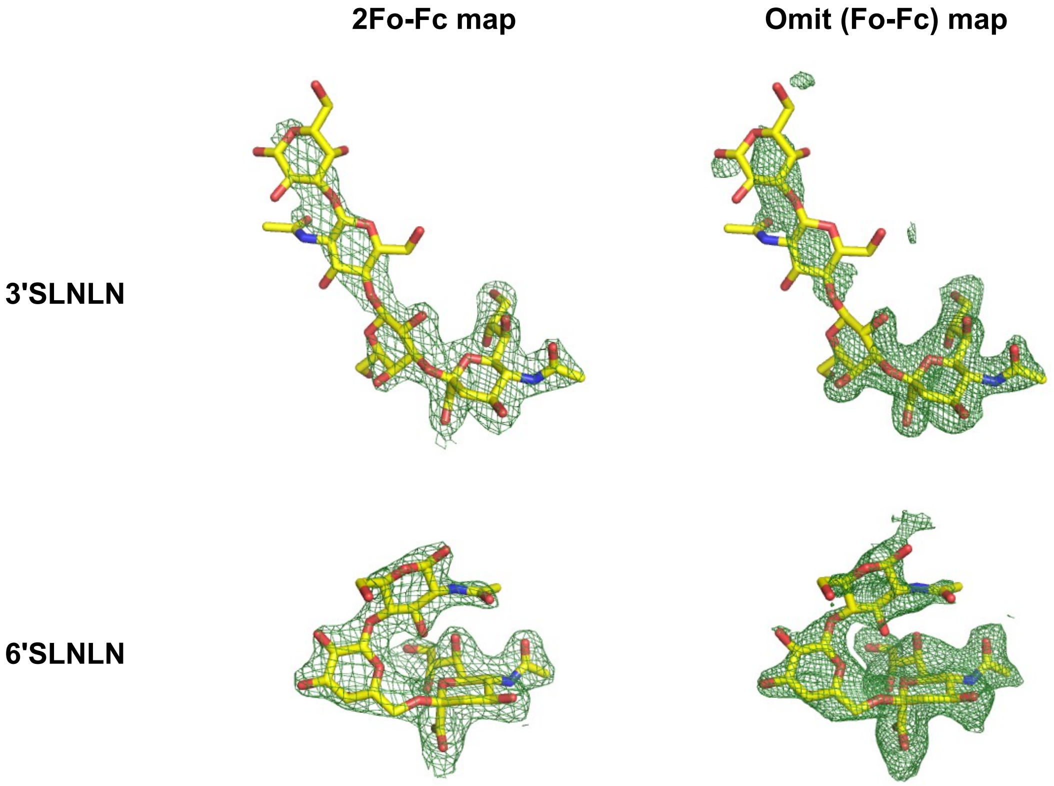

Figure S2. Final 2Fo-Fc and omit (Fo-Fc) electron density maps of glycan receptor analogs (3'SLNLN or 6'SLNLN), Related to Figure 4. Final 2Fo-Fc electron density maps for the glycan receptor analogs (yellow sticks) are represented in a green mesh and contoured at 0.8 σ (left). Omit (Fo-Fc) electron density maps for the glycan receptor analogs are represented in a green mesh and contoured at 2.0 σ (right).

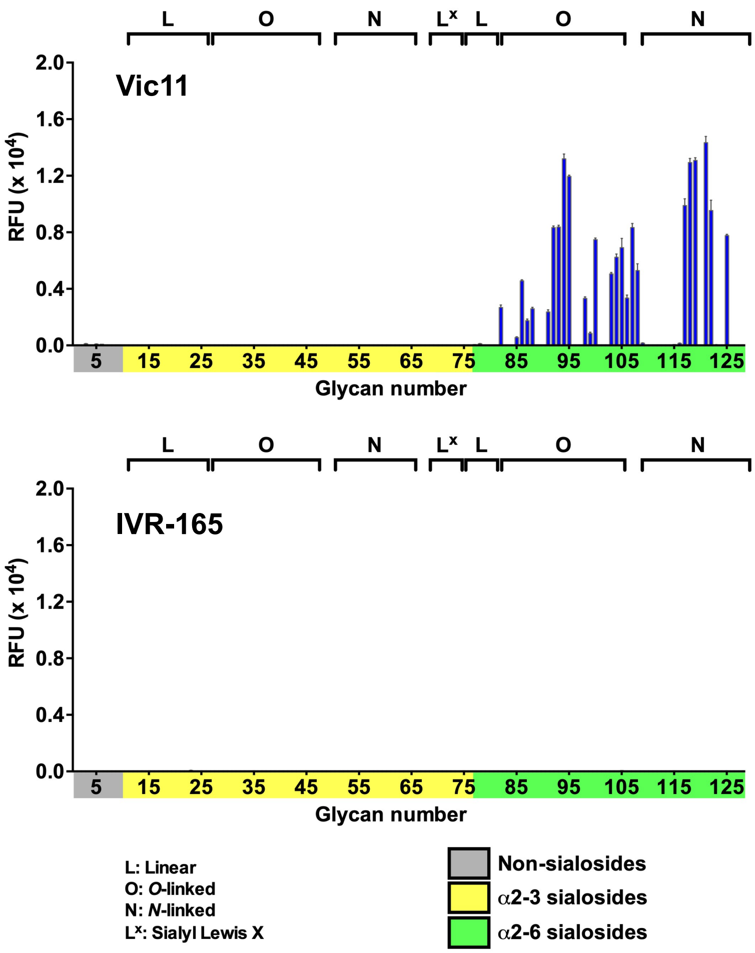

Figure S3. Glycan array analysis of recombinant HA from Vic11 and IVR-165, Related to Figure 4. 293S-expressed recombinant HA was purified and analyzed on the sialoside glycan array.

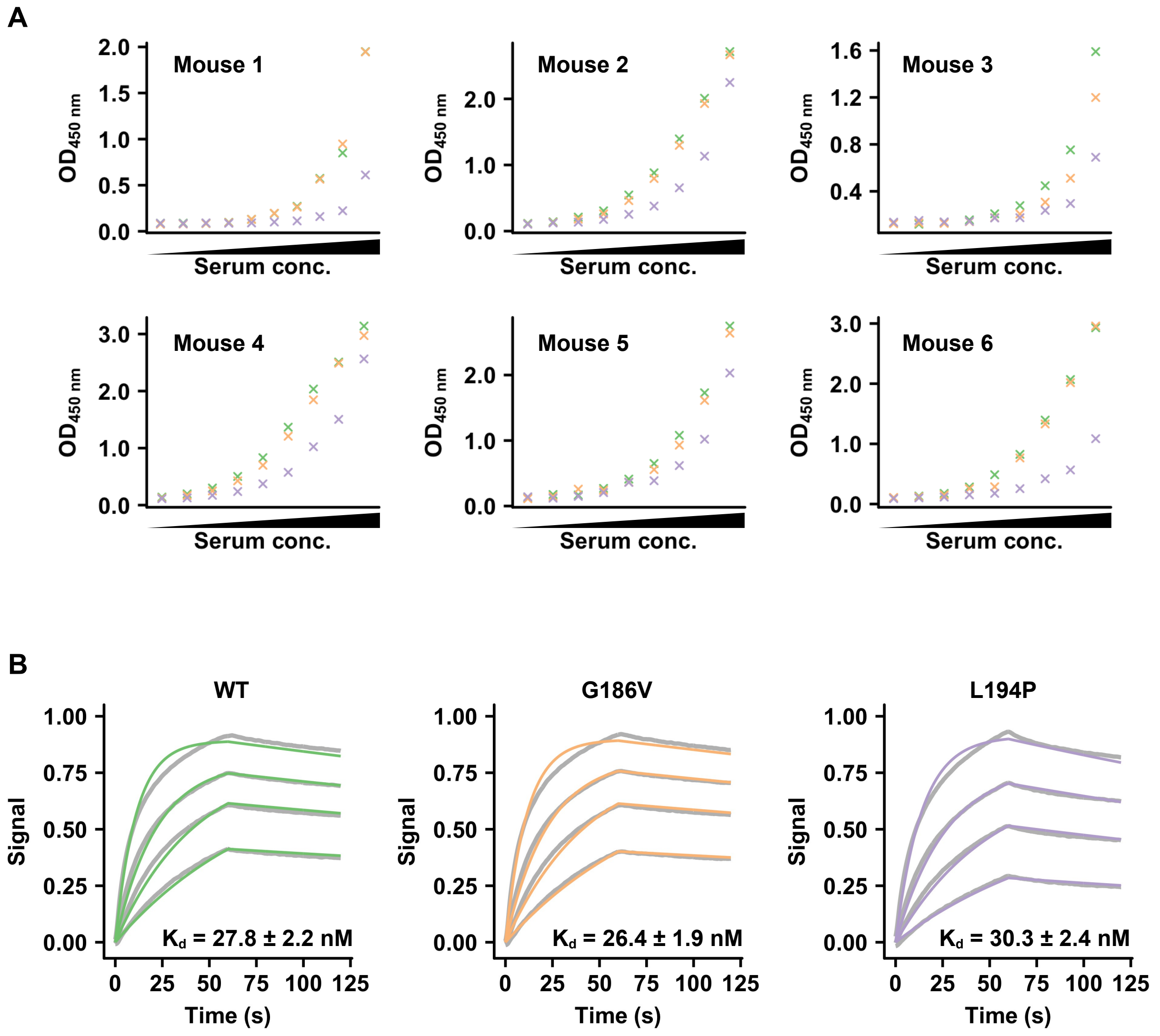

Figure S4. Binding of mouse sera and CR9114 to Bris07 WT or mutant HA, Related to Figure 6.

(A) A total of 6 mice were immunized with Bris07 WT virus (6:2 reassortant on a PR8 backbone). Sera from immunized mice were tested for binding to WT (green), G186V mutant (orange), and L194P mutant (purple) of recombinant Bris07 HA using ELISA. X-axis represents the 2-fold serial dilution of serum at different concentrations (right: highest tested concentration, left: lowest tested concentration). The highest tested concentration was 1:500 dilution of the serum sample (the second highest tested concentration was 1:1000 dilution of the serum sample, and so on).

(B) Biolayer interferometry (BLI) was used to measure the binding kinetics of CR9114 IgG against the recombinant HAs of Bris07 WT, G186V mutant, and L194P mutant. Grey lines represent the response curve and colored lines represent the 1:1 binding model.
