# Supplementary material for: Preventing an Antigenically Disruptive Mutation in Egg-Based H3N2 Seasonal Influenza Vaccines by Mutational Incompatibility
Source: Cell Host Microbe. 2019 Jun 12;25(6):836–844.e5. doi: 10.1016/j.chom.2019.04.013 (PMC6579542; doi:10.1016/j.chom.2019.04.013)
Supplement: Document S2. Article plus Supplemental Information [file mmc3.pdf]

Article

Cell Host & Microbe

Preventing an Antigenically Disruptive Mutation in Egg-Based H3N2 Seasonal Influenza Vaccines by Mutational Incompatibility

Graphical Abstract

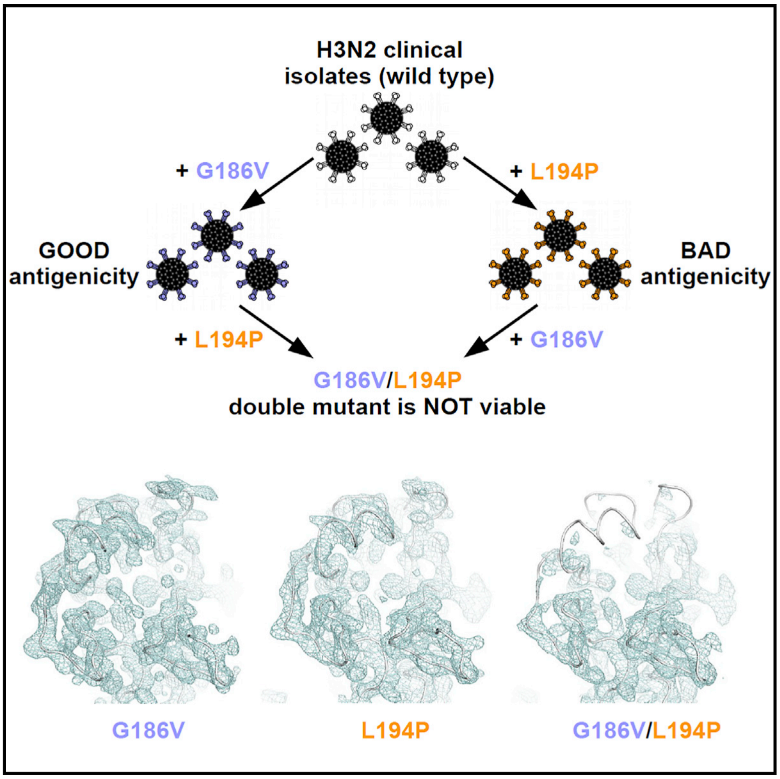

Highlights

- Most H3N2 egg isolates carry hemagglutinin mutation G186V or L194P, but not both
- Hemagglutinin double mutation G186V/L194P is highly deleterious to the virus
- Hemagglutinin double mutation G186V/L194P disrupts the receptor-binding site
- Wild-type hemagglutinin antigenicity is maintained in G186V, but not in L194P

Authors

Nicholas C. Wu, Huibin Lv, Andrew J. Thompson, ..., James C. Paulson, Chris K.P. Mok, Ian A. Wilson

Correspondence

ch02mkp@hku.hk (C.K.P.M.), wilson@scripps.edu (I.A.W.)

In Brief

Wu et al. characterize the incompatibility between two major egg-adaptive mutations in human H3N2 virus hemagglutinin, G186V and L194P, which confer either minimal or significant antigenic change, respectively. This study suggests that the antigenically disruptive mutation L194P that occurs during egg-based influenza vaccine production can be prevented by mutational incompatibility.

Wu et al., 2019, Cell Host & Microbe *25*, 836–844
June 12, 2019 © 2019 The Authors. Published by Elsevier Inc.
https://doi.org/10.1016/j.chom.2019.04.013

CellPress

Cell Host & Microbe
Article

Preventing an Antigenically Disruptive Mutation in Egg-Based H3N2 Seasonal Influenza Vaccines by Mutational Incompatibility

Nicholas C. Wu,[1] Huibin Lv,[2,3] Andrew J. Thompson,[4] Douglas C. Wu,[5,6] Wilson W.S. Ng,[2,3] Rameshwar U. Kadam,[1] Chih-Wei Lin,[7] Corwin M. Nycholat,[4] Ryan McBride,[4] Weiwen Liang,[3] James C. Paulson,[4,8] Chris K.P. Mok,[2,3,*] and Ian A. Wilson[1,9,10,*]

[1]Department of Integrative Structural and Computational Biology, The Scripps Research Institute, La Jolla, CA 92037, USA
[2]HKU-Pasteur Research Pole, School of Public Health, Li Ka Shing Faculty of Medicine, The University of Hong Kong, Hong Kong SAR, China
[3]State Key Laboratory of Respiratory Disease, National Clinical Research Center for Respiratory Disease, Guangzhou Institute of Respiratory Health, the First Affiliated Hospital of Guangzhou Medical University, Guangzhou, Guangdong 510000, China
[4]Department of Immunology and Microbiology, The Scripps Research Institute, La Jolla, CA 92037, USA
[5]Institute for Cellular and Molecular Biology, The University of Texas at Austin, Austin, TX 78712, USA
[6]Department of Molecular Biosciences, The University of Texas at Austin, Austin, TX 78712, USA
[7]Department of Chemistry, The Scripps Research Institute, La Jolla, CA 92037, USA
[8]Department of Molecular Medicine, The Scripps Research Institute, La Jolla, CA 92037, USA
[9]The Skaggs Institute for Chemical Biology, The Scripps Research Institute, La Jolla, CA 92037, USA
[10]Lead Contact
*Correspondence: ch02mkp@hku.hk (C.K.P.M.), wilson@scripps.edu (I.A.W.)
https://doi.org/10.1016/j.chom.2019.04.013

SUMMARY

Egg-based seasonal influenza vaccines are the major preventive countermeasure against influenza virus. However, their effectiveness can be compromised when antigenic changes arise from egg-adaptive mutations on influenza hemagglutinin (HA). The L194P mutation is commonly observed in egg-based H3N2 vaccine seed strains and significantly alters HA antigenicity. An approach to prevent L194P would therefore be beneficial. We show that emergence of L194P during egg passaging can be impeded by preexistence of a G186V mutation, revealing strong incompatibility between these mutations. X-ray structures illustrate that individual G186V and L194P mutations have opposing effects on the HA receptor-binding site (RBS), and when both G186V and L194P are present, the RBS is severely disrupted. Importantly, wild-type HA antigenicity is maintained with G186V, but not L194P. Our results demonstrate that these epistatic interactions can be used to prevent the emergence of mutations that adversely alter antigenicity during egg adaptation.

INTRODUCTION

Seasonal influenza vaccines offer protection against two influenza A virus subtypes, H1N1 and H3N2, as well as against influenza B virus. However, the effectiveness of seasonal influenza vaccines has been disappointing, especially for H3N2 viruses (Belongia et al., 2016). Their low effectiveness, other than occasional virus mismatch, can be attributed to the egg-based production process (Raymond et al., 2016; Skowronski et al., 2014; Wu et al., 2017b; Zost et al., 2017). Influenza virus hemagglutinin (HA) engages the host sialylated glycan receptor at the receptor-binding site (RBS) to initiate the virus life cycle. Growth of the virus in eggs occurs in the chorioallantoic membrane, which contains sialylated glycans that are short and are predominantly α2-3 linked (avian-type receptors) (Sriwilaijaroen et al., 2009). In contrast, sialylated glycans in human ciliated tracheal epithelial cells, which are the natural host cells for human influenza virus, are predominantly α2-6 linked (human-type receptors) (Couceiro et al., 1993). Furthermore, the receptor specificity of recent human H3N2 viruses has evolved to be mainly for long, branched α2-6 sialylated glycans (Peng et al., 2017). There is also a strong selection pressure for human H3N2 viruses to acquire mutations in the HA RBS during passaging in eggs to adapt back to the avian-type receptors present in the chorioallantoic membrane. Since the HA RBS partially overlaps with several major antigenic sites (Wiley et al., 1981; Wilson et al., 1981; Wu and Wilson, 2017), egg-adaptive mutations can dramatically alter HA antigenicity and reduce the effectiveness of seasonal influenza vaccines (Raymond et al., 2016; Skowronski et al., 2014; Wu et al., 2017b; Zost et al., 2017). While such problems can be resolved by cell-based or recombinant influenza vaccines, most influenza vaccines available on the market remain egg based because of low production cost and existing infrastructure for their annual production (Harding and Heaton, 2018).

Several egg-adaptive mutations on the HA of human H3N2 viruses, such as H156Q (Jin et al., 2005; Skowronski et al., 2014), L194P (Chen et al., 2010b; Popova et al., 2012; Wu et al., 2017b), and T160K (loss of glycosylation) (Zost et al., 2017), have been shown to affect antigenicity. However, other egg-adaptive HA mutations on human H3N2 viruses, such as H183L (Lu et al., 2005), G186V (Barman et al., 2015; Lu et al., 2006, 2005; Parker et al., 2016; Widjaja et al., 2006), A196T (Lu et al., 2006), S219F (Widjaja et al., 2006), V226A (Lu et al., 2005), and V226I (Lu et al.,

 © 2019 The Authors. Published by Elsevier Inc.
This is an open access article under the CC BY license (http://creativecommons.org/licenses/by/4.0/).

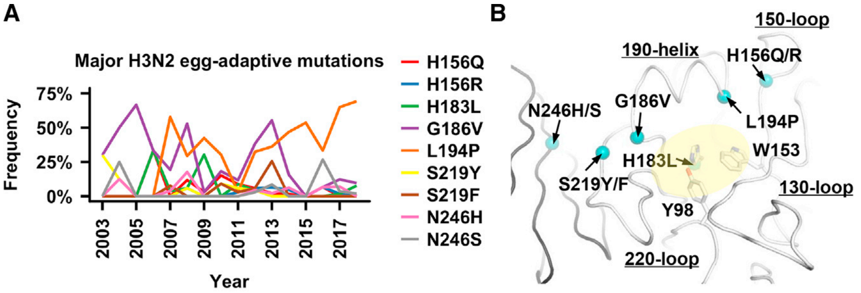

C

H3N2 strain	HA residue					
	156	183	186	194	219	246
A/Wisconsin/67/2005 (NYMC X-161)	Q	H	V	L	S	N
A/Brisbane/10/2007 (NYMC X-171)	H	H	V	L	S	N
A/Brisbane/10/2007 (IVR-147)	H	H	G	P	S	N
A/Uruguay/716/2007 (NYMC X-175C)	H	H	G	P	S	N
A/Victoria/210/2009 (NYMC X-187)	H	H	V	L	S	N
A/Victoria/361/2011 (IVR-165)	Q	H	V	L	Y	N
A/Texas/50/2012 (NYMC X-223A)	H	H	V	L	F	N
A/South Australia/55/2014 (IVR-175)	R	H	E	L	S	N
A/Hong Kong/4801/2014 (NYMC X-263B)	H	H	G	P	S	N
A/Singapore/INFIMH-16-0019/2016 (NIB-104)	H	H	G	P	S	N
A/Singapore/INFIMH-16-0019/2016 (IVR-186)	H	H	G	P	S	N
A/Switzerland/8060/2017 (NIB-112)	H	H	G	P	S	N

D

H3N2 strain	HA residue					
	156	183	186	194	219	246
A/Kansas/14/2017 (IVR-195)	H	H	V	L	Y	N
A/Kansas/14/2017 (X-327)	H	H	V	L	Y	N
A/Kansas/14/2017 (CBER-22C)	H	H	V	L	Y	N
A/Kansas/14/2017 (CBER-22B/CDC19A)	H	H	V	L	Y	N

Figure 1. Egg-Adaptive Mutations in Human H3N2 Virus

(A) Occurrence frequencies of seven major egg-adaptive mutations in egg-passaged human isolates from different years are shown. Mutations are numbered according to H3 numbering. The absolute residue numberings (from the first Met in the HA protein as 1) for residues 156, 183, 186, 194, 219, and 246, are 172, 199, 202, 210, 235, and 262, respectively.

(B) The Cα positions of the major egg-adaptive mutations are shown on the HA structure as teal spheres. The binding site for the sialic acid is shaded by a yellow oval.

(C) For each of the nine representative vaccine strains listed in Table S2, the amino-acid identities at residues 156, 183, 186, 194, 219, and 246 (H3 numbering) are shown. Egg-adaptive mutations are highlighted in yellow. Of note, the egg-adaptive mutation G186E in A/South Australia/55/2014 (IVR-175) was seldom observed during egg adaptation. Two egg-adapted strains that were derived from A/Brisbane/10/2007, namely NYMC X-171 (GISAID: EPI_ISL_23300) and IVR-147 (GISAID: EPI_ISL_20693), are also included here for comparison.

(D) A/Kansas/14/2017 is the H3N2 vaccine strain for the 2019–2020 northern hemisphere influenza season. The amino-acid identities at residues 156, 183, 186, 194, 219, and 246 are shown for four egg-adapted strains that were derived from A/Kansas/14/2017. The sequences were downloaded from GISAID with the following accession numbers: IVR-195 (GISAID: EPI_ISL_346482), X-327 (GISAID: EPI_ISL_346457), CBER-22C (GISAID: EPI_ISL_346456), and CBER-22B/CDC19A (GISAID: EPI_ISL_346455). Egg-adaptive mutations are highlighted in yellow.

2005; Widjaja et al., 2006), minimally impact antigenicity. While egg-adaptive mutations are critical for high-yield production of egg-based seasonal influenza vaccines, the ideal egg-based vaccine should only carry those egg-adaptive mutations that minimally affect antigenicity. As egg-based vaccines are likely to remain the major global preventive measures against seasonal influenza viruses in the foreseeable future, it is important to characterize the evolution of the influenza virus during egg adaptation.

Based on sequence database analysis and virus rescue experiments, this study revealed that the two most common egg-adaptive mutations on H3N2 HA, namely G186V and L194P, are incompatible. In other words, the HA G186V/L194P double mutant was not viable despite each of the single mutants being viable. Passaging the G186V mutant in eggs could prevent the emergence of the L194P mutation and vice versa. Structural analysis illustrated that mutations G186V and L194P had opposing structural effects. The relative height of the HA RBS is increased by G186V but decreased by L194P. This structural variation between G186V and L194P then leads to differences in the receptor-binding modes. When mutations G186V and L194P are both present, the HA RBS is disrupted, explaining the incompatibility of G186V and L194P. Consistent with previous studies (Barman et al., 2015; Chen et al., 2010b; Lu et al., 2006, 2005; Parker et al., 2016; Popova et al., 2012; Widjaja et al., 2006; Wu et al., 2017b), we also showed that G186V has minimal antigenic effect, whereas L194P strongly impacts antigenicity. In summary, this study reveals and characterizes two mutually exclusive evolutionary trajectories for egg adaptation of human H3N2 viruses, which provides important insights into the seed-strain selection for egg-based seasonal influenza vaccines.

RESULTS

Major Egg-Adaptive Mutations from Sequence Database

Egg-adaptive mutations can be readily observed after passaging human H3N2 clinical isolates in embryonated chicken eggs. To identify such mutations and ascertain their prevalence, HA protein sequences of influenza clinical isolates along with their passaging history were obtained from Global Initiative for Sharing Avian Influenza Data (GISAID; http://gisaid.org). H3 numbering is used in this study. Nine mutations, namely H156Q, H156R, H183L, G186V, L194P, S219Y, S219F, N246H, and N246S, were classified as the major egg-adaptive mutations (see STAR Methods; Figures 1A and 1B; Table S1). These observations are consistent with previous studies, which showed that growth of the H3N2 virus in eggs can be enhanced by H183L (Lu et al., 2005), G186V (Barman et al., 2015; Lu et al., 2005; Stevens et al., 2010; Widjaja et al., 2006), L194P (Chen et al., 2010b;

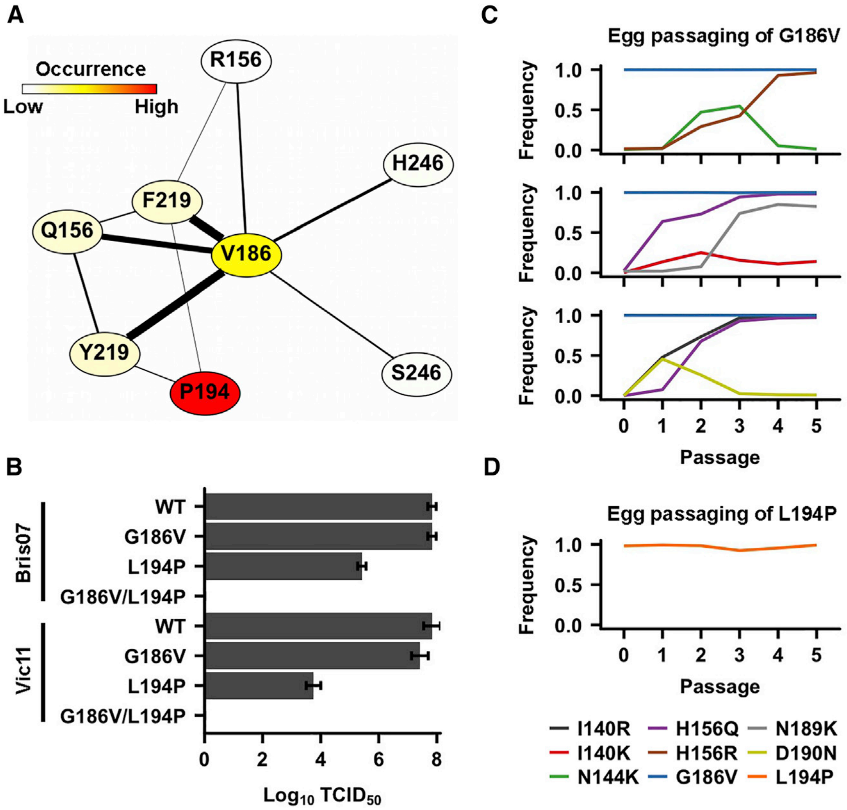

Figure 2. Incompatibility of Egg-Adaptive Mutations HA L194P and G186V

(A) A network diagram was constructed that describes the co-occurrence of egg-adaptive mutations and was based on the sequence analysis of egg-passaged human H3N2 viruses in the GISAID database. Each node represents an egg-adaptive mutation. Two mutations that co-occur more than once are connected by an edge. The width of the edge is proportional to the co-occurrence frequency. The occurrence frequency of each egg-adaptive mutation is color coded. L183 is not shown because it does not co-occur with other major egg-adaptive mutations in more than one isolate.

(B) The fitness effects of different mutants were examined by a virus rescue experiment. The titer was measured by median tissue culture infectious dose ($TCID_{50}$). Error bars indicate the standard deviation of three independent experiments.

(C and D) Bris07 HA G186V mutant virus (C) and Bris07 HA L194P mutant virus (D) were passaged for five rounds in eggs. The emergence of egg-adaptive mutations in the receptor-binding domain (HA1 residues 117–265) was monitored by next-generation sequencing. Only those mutations that reached a minimum of 10% occurrence frequency are plotted. Three independent passaging experiments were performed for the Bris07 HA G186V mutant virus in (C) and one passaging experiment was performed for the Bris07 HA L194P mutant virus in (D). Passage 0 indicates the input virus.

Hartgroves et al., 2010; Stevens et al., 2010), S219Y (Meyer et al., 1993; Parker et al., 2016; Stevens et al., 2010), S219F (Parker et al., 2016; Widjaja et al., 2006), and loss of a glycosylation site at position 246 (Barman et al., 2015). These seven major egg-adaptive mutations are all located within or adjacent to the HA RBS (Figure 1B). Among them, G186V and L194P, which are located on opposite sides of the 190-helix, had particularly high-occurrence frequencies (Figure 1A).

We further examined the protein sequences of nine H3N2 vaccine seed strains that were used for egg-based influenza vaccine production (Table S2). Six of the nine major egg-adaptive mutations, H156Q, H156R, G186V, L194P, S219Y, and S219F, could be found in the vaccine seed strains (Figure 1C). The most commonly observed egg-adaptive mutations among these nine vaccine seed strains were G186V and L194P, each of which was carried by four vaccine seed strains. However, none of the nine vaccine seed strains carried both G186V and L194P.

The H3N2 vaccine seed stock for the 2009–2010 northern hemisphere influenza season was derived from A/Uruguay/716/2007, which was antigenically equivalent to A/Brisbane/10/2007 (Bris07). A/Uruguay/716/2007 (NYMC X-175C) carried an egg-adaptive mutation, L194P, which has been extensively characterized, both structurally and antigenically, in our previous study using Bris07 HA (Wu et al., 2017b). Interestingly, two egg-adapted strains that were derived from Bris07, namely NYMC X-171 and IVR-147, carried different egg-adaptive mutations (Figure 1C). While NYMC X-171 carried mutation G186V but not L194P, IVR-147 carried mutation L194P but not G186V. Recently, the World Health Organization (WHO) announced A/Kansas/14/2017 (Kansas17) as the H3N2 vaccine strain for the 2019–2020 northern hemisphere influenza season. Therefore, we also examined the sequences of four egg-adapted strains that were derived from Kansas17 (Figure 1D). Consistent with our observations above, these four strains carried G186V but not L194P. Overall, this analysis demonstrates the potential clinical relevance of the egg-adaptive mutations and further suggests that G186V and L194P represent two distinct evolutionary pathways for egg adaptation.

Incompatibility of G186V and L194P

Next, we aimed to investigate whether there was any relationship between G186V and L194P. Based on the egg-passaged human H3N2 virus in the GISAID database, a network diagram was constructed to visualize the co-occurrence of the nine major egg-adaptive mutations (Figures 2A; see STAR Methods). In this network diagram, each node represents an egg-adaptive mutation. Co-occurring mutations are connected by an edge. While G186V and L194P are the most commonly observed egg-adaptive mutations in H3N2, they did not co-occur in the same virus. We also performed a virus rescue experiment (Figure 2B). Both G186V and L194P single mutants could be rescued to a reasonable titer despite the lower titer of the L194P mutant. In contrast, the G186V/L194P double mutant could not be rescued. This result is consistent between two H3N2 genetic backgrounds: namely A/Victoria/361/2011 (Vic11) and Bris07. In fact, a previous study also failed to rescue the G186V/L194P double mutant in Bris07, as well as in another human H3N2 strain A/Wisconsin/67/05 (Chen et al., 2010b). Overall, our analyses show that G186V and L194P are incompatible.

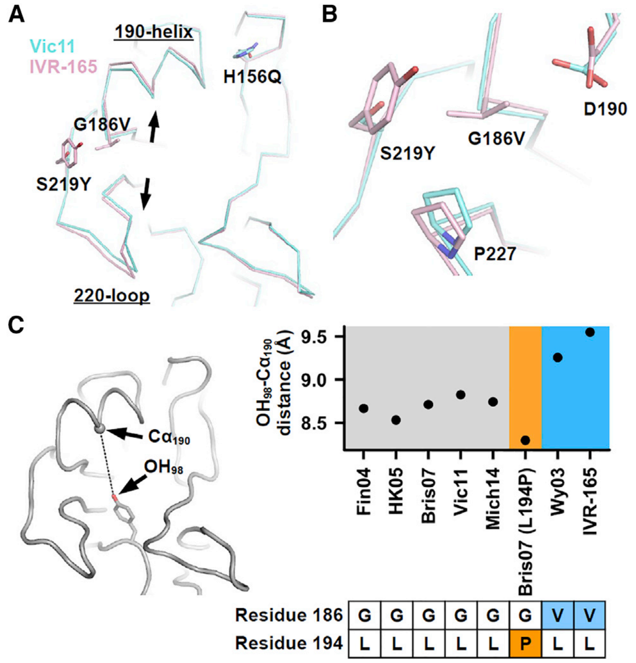

Figure 3. Structural Comparison of IVR-165 HA and Vic11 HA

(A) The HA RBS conformations of IVR-165 HA and Vic11 HA were compared by aligning their receptor-binding subdomains (HA1 residues 117–265). The slight expansion of the RBS due to the backbone shifts of the 190-helix and 220-loop is indicated by the arrows. IVR-165 HA that was used for structural determination was expressed recombinantly in insect cells (see STAR Methods).

(B) A zoomed-in view of the RBS backbone shift.

(C) The distances between the phenolic oxygen of Tyr 98 (OH_{98}) and the Cα of residue 190 ($C\alpha_{190}$) in different H3 strains were measured: Wy03: PDB 6BKN (Wu et al., 2018), Fin04: PDB 2YP2 (Lin et al., 2012), HK05: PDB 2YP7 (Lin et al., 2012), Bris07: PDB 6AOR (Wu et al., 2017b), Bris07 (L194P): PDB 6AOP (Wu et al., 2017b), Vic11: PDB 4O5N (Lee et al., 2014), and Mich14: PDB 6BKP (Wu et al., 2018).

We postulated that since G186V and L194P are mutually exclusive, the evolutionary trajectories of the H3N2 virus in eggs would be influenced by whichever mutation (i.e., G186V or L194P) appears first. We then performed an experiment by passaging the Bris07 G186V mutant to monitor the emergence of any mutations in the HA receptor-binding subdomain (HA1 residue 117–265) using next-generation sequencing. The G186V mutant was passaged in eggs in triplicate (Figure 2C). As expected, when the G186V mutant was passaged in eggs, the L194P mutation did not emerge. Instead, mutations I140K, I140R, N144K, H156R, H156Q, N189K, and D190N were observed with a frequency of >10% in any passage, on the background of G186V. In comparison, when the L194P mutant was passaged in eggs, no mutation was able to reach a frequency of >10% (Figure 2D). Overall, these results demonstrate that the evolutionary trajectories during H3N2 egg adaptation of G186V and L194P mutants are different, corroborating the incompatibility of G186V and L194P.

G186V Increases the Height of the HA RBS

Previously, we performed a thorough structural study of the L194P mutation (Wu et al., 2017b). To dissect the impact of the G186V mutation, a crystal structure of IVR-165 HA, which is the triple mutant H156Q/G186V/S219Y of Vic11 HA, was determined at a resolution of 2.25 Å (Table S3). IVR-165 was the vaccine seed strain for the 2012–2013 influenza season (Table S2). The HA structure of IVR-165 was compared with that of Vic11 by aligning their receptor-binding subdomains (Figures 3A and 3B). The distance between the 190-helix and 220-loop in IVR-165 HA RBS is slightly larger than that in the Vic11 HA RBS. Such a difference can be attributed to the G186V mutation, which causes the 190-helix to move away from the 220-loop through an increase in the side-chain volume. The other two egg-adaptive mutations H156Q and S219Y have little, if any, influence on the distance between the 190-helix and 220-loop. H156Q abolishes a hydrogen bond between the 150-loop and 190-helix (Figures S1A and S1B), whereas S219Y stabilizes the N165 glycan from the neighboring promoter of the trimer by forming a stacking interaction with the glycan (Figure S1C). Therefore, the observed distance increase between the 190-helix and 220-loop is mainly a result of the G186V mutation.

We further compared the height of the HA RBS of IVR-165 with that of other H3 strains (Figure 3C), namely A/Wyoming/3/2003 (Wy03) (Wu et al., 2018), A/Finland/486/2004 (Fin04) (Lin et al., 2012), A/Hong Kong/4443/2005 (HK05) (Lin et al., 2012), Bris07 (Wu et al., 2017b), Vic11 (Lee et al., 2014), and A/Michigan/15/2014 (Mich14) (Wu et al., 2018). Besides IVR-165 HA, Wy03 HA also carried the G186V mutation. The heights of the RBS of Wy03 and IVR-165, as measured by the distance between OH_{98} and $C\alpha_{190}$, are 9.3 and 9.5 Å, respectively. In comparison, those equivalent distances of Fin04, HK05, Bris07, Vic11, and Mich14 are in the range of 8.5–8.8Å. Overall, our structural analysis indicates that G186V increases the height and, hence, the size of the HA RBS.

G186V and L194P Lead to Differences in Receptor-Binding Mode

To understand how G186V influences receptor binding, crystal structures of IVR-165 HA in complex the with avian receptor analog NeuAcα2-3Galβ1-4GlcNAcβ1-3Galβ1-4GlcNAc (3′SLNLN) and the human receptor analog NeuAcα2-6Galβ1-4GlcNAcβ1-3Galβ1-4GlcNAc (6′SLNLN) were determined at 2.1 and 2.4 Å, respectively (Table S3; Figure S2). When binding to IVR-165 HA, 3′SLNLN adopts an extended conformation (Figure 4A), whereas 6′SLNLN adopts the canonical folded-back conformation (Figure 4B). We also observed a shift of the 220-loop toward the RBS during receptor binding (Figures 4A and 4B), which is a typical feature of recent human H3 viruses (Lin et al., 2012). Similar to our previous observation with Bris07 L194P HA (Wu et al., 2017b), IVR-165 HA had negligible binding to the glycan array (Figure S3). Of note, the glycan array has more stringent requirements for the detection of receptor binding than the X-ray structure analysis here, which involves soaking the crystals in a 100-fold molar excess of the receptor analogs. Being able to observe electron density for the receptor analogs in our crystal structures but no signal in the glycan array experiments indicates very weak binding to the receptor. This result also substantiates the notion that efficient replication of human influenza virus in chicken eggs does not require strong binding between HA and sialylated receptors (Wu et al., 2017b).

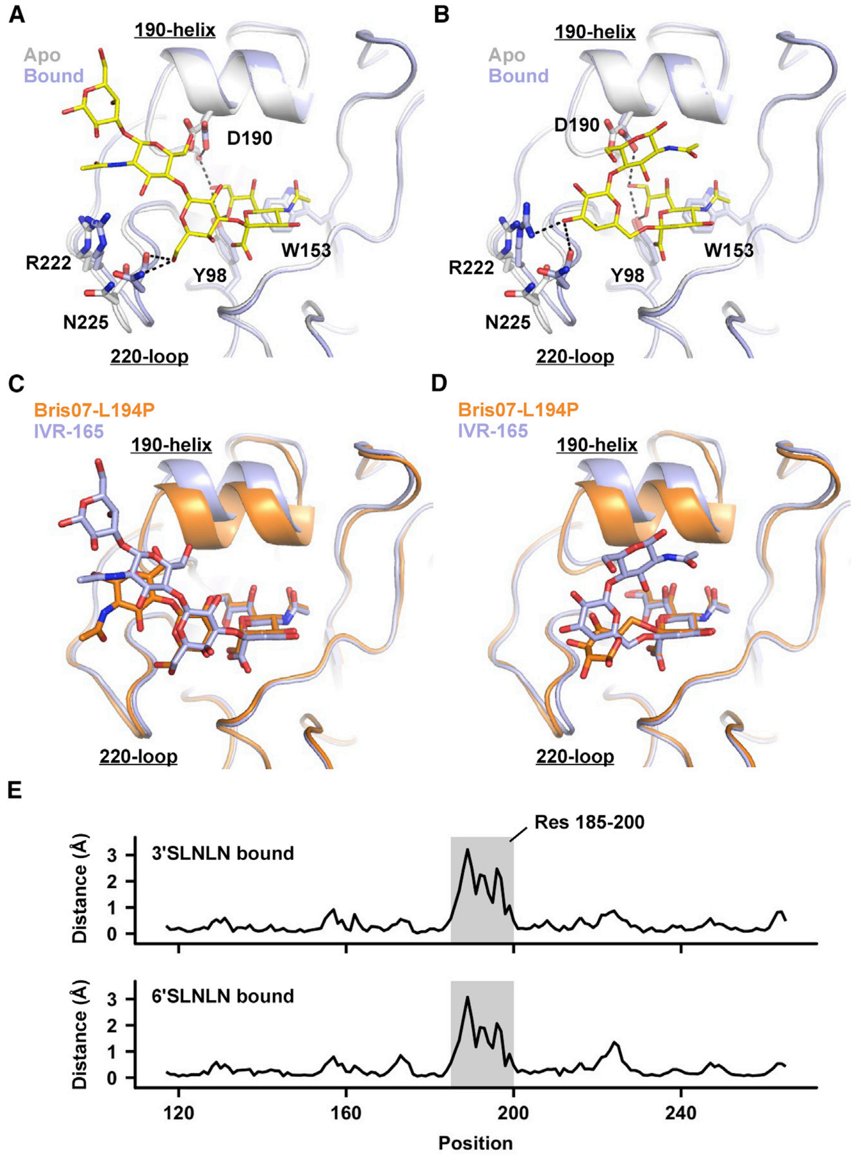

Figure 4. Structural Analysis of IVR-165 HA in Complex with Glycan Receptor Analogs

(A and B) HA crystal structures of IVR-165 in complex with 3′SLNLN (A) and IVR-165 in complex with 6′SLNLN (B). The apo form is aligned on the complexes (blue) and colored in whitish gray. Glycan receptor analogs (3′SLNLN and 6′SLNLN) are colored in yellow and shown in stick representation. Hydrogen bonds are represented by black dashed lines.

(C) The HA RBS conformations of IVR-165 HA and Bris07-L194P HA in complex with 3′SLNLN are compared. Structural alignment here and in (D) was performed using the receptor-binding subdomain (HA1 residues 117–265).

(D) The RBS conformations of IVR-165 HA and Bris07 HA-L194P in complex with 6′SLNLN are compared.

(E) The distance between equivalent Cαs of IVR-165 and Cαs of Bris07-L194P for each residue in the receptor-binding subdomain (HA1 residues 117–265) is shown. This analysis was performed on the structural alignment of IVR-165 and Bris07-L194P in complex with 3′SLNLN (upper panel) and in complex with 6′SLNLN (bottom panel). The major differences are seen in the 190-helix (shaded region).

Interestingly, noticeable differences can be observed between the receptor-binding modes of IVR-165 HA and that of the Bris07 HA L194P mutant (Wu et al., 2017b) when their receptor-binding subdomains (HA1 residues 117–265) are aligned (Figures 4C and 4D). GlcNAc-3 of the 3′SLNLN in complex with the Bris07 HA L194P mutant shifts away from the RBS as compared to that of 3′SLNLN in complex with IVR-165 HA (Figure 4C). For 6′SLNLN, Gal-2 is rotated by 90° when binding to Bris07 HA L194P (Wu et al., 2017b), as compared to the folded-back conformation when binding to IVR-165 HA (Figure 4D). Such differences in receptor binding can be attributed to the difference in the positioning of the 190-helix, i.e., the height of the RBS. In contrast to mutation G186V, which increases the RBS height, mutation L194P appears to decrease its height (Figure 3C). The distance between OH_{98} and $C\alpha_{190}$ of the Bris07 HA L194P mutant is 8.3 Å, whereas that of Bris07 wild-type (WT) HA is 8.7 Å. We further measured the difference in backbone conformations between the receptor-binding subdomains of Bris07 HA and IVR-165 HA in their receptor-bound states (Figure 4E). This analysis reveals that shifts in the polypeptide backbone between Bris07 HA and IVR-165 HA in the 190-helix can be as large as 3 Å. Consequently, the difference in receptor-binding mode between IVR-165 HA and the Bris07 HA L194P mutant can be explained by the opposing structural effects of the G186V and L194P mutations.

HA G186V/L194P Double Mutant Disrupts the RBS

With the knowledge that HA mutations G186V and L194P exert an opposing structural effect on the RBS (Figures 3C, 4C, and 4D) and that the HA G186V/L194P double mutant was highly deleterious to the virus (Figure 2B), we were interested in examining the structural effect of the HA G186VL194P double mutant. Of note, the HA G186V/L194P double mutant abolishes virus replication but not HA protein expression. We were therefore able to recombinantly express and purify the Bris07 HA G186V/L194P double mutant. Crystal structures of the Bris07 HA G186V/L194P double mutant were determined at 2.25 Å, 2.1 Å, and 2.4 Å for the apo form, in complex with 3′SLNLN, and in complex with 6′SLNLN, respectively. In all three structures, the electron density for the 190-helix was very weak to absent compared to that of the Bris07 HA L194P single mutant (Figure 5), whose structure was previously determined in the same crystallization condition (Wu et al., 2017b). This observation indicates that the 190-helix in Bris07 HA G186V/L194P double mutant is extremely disordered. Consistently, the electron density for the receptor analogs 3′SLNLN and 6′SLNLN was also very weak in the complex with

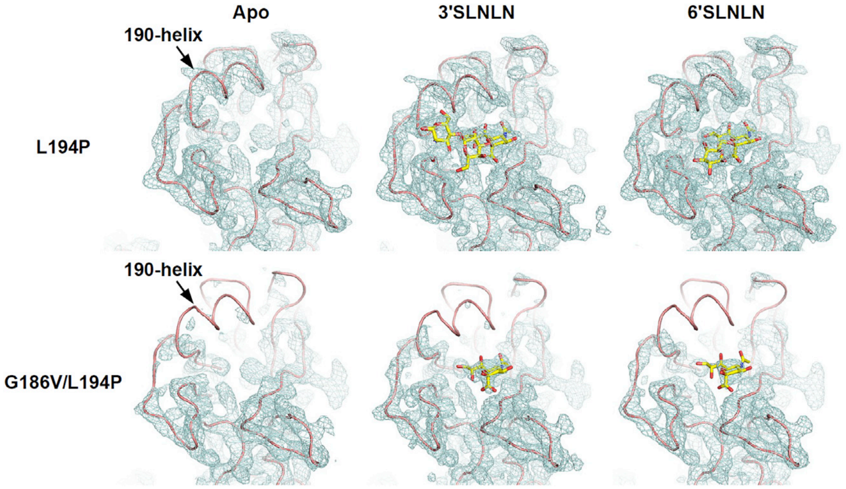

Figure 5. Electron Density of the 190-Helix

Final 2Fo-Fc electron density maps for the HA receptor-binding site of the L194P mutant (top) and G186V/L194P double mutant (bottom) are represented in a blue mesh and contoured at 0.8 σ. Apo form (left panel), complex with 3′SLNLN (middle panel), and complex with 6′SLNLN (right panel). Final 2Fo-Fc electron density maps and coordinates for L194P mutant were retrieved from PDB 6AOP (apo form), PDB 6AOS (in complex with 3′SLNLN), and PDB 6AOT (in complex with 6′SLNLN) (Wu et al., 2017b). The HA of the Bris07 G186V/L194P double mutant was expressed recombinantly in insect cells (see STAR Methods).

the Bris07 HA G186V/L194P double mutant. This structural analysis shows that combining HA mutations G186V and L194P dramatically destabilizes the 190-helix, causing a disruption of the RBS and also of antigenic site B, which is the major antigenic site in recent H3N2 viruses (Broecker et al., 2018; Popova et al., 2012).

G186V Imposes Minimum Change in Antigenicity

Previously, we and others demonstrated that the HA antigenicity is significantly altered by the L194P mutation (Chen et al., 2010b; Popova et al., 2012; Wu et al., 2017b), when using ferret sera (Chen et al., 2010b), human sera (Wu et al., 2017b), and human monoclonal antibodies (Popova et al., 2012; Wu et al., 2017b). Furthermore, based on a ferret study, L194P has been shown to significantly decrease virus immunogenicity (Chen et al., 2010b). In contrast, the G186V mutation has been shown to confer minimal HA antigenic differences (Barman et al., 2015; Lu et al., 2006; Lu et al., 2005; Parker et al., 2016; Widjaja et al., 2006), using ferret sera (Barman et al., 2015; Lu et al., 2006; Lu et al., 2005; Parker et al., 2016), sheep sera (Widjaja et al., 2006), and mouse monoclonal antibodies (Widjaja et al., 2006). Sera from ferrets that were immunized with a virus that carried the G186V mutation cross-react well with the WT virus, with only a 2-fold decrease in hemagglutination inhibition (HAI) titer when compared to the sera from ferrets that were immunized with the WT virus (Barman et al., 2015). Here, we further performed a side-by-side comparison of the HA antigenic changes resulting from the G186V and L194P mutations. Sera from six mice that were immunized with unpassaged Bris07 WT virus (6:2 reassortant on PR8 backbone) were obtained. The immunization scheme consisted of infection of BALB/c mice with a non-adjuvanted virus followed by a boost with an Addavax-adjuvanted virus. An ELISA experiment was then performed to assess binding of these sera to Bris07 WT, G186V, and L194P recombinant HA proteins (Figures 6A and S4A). For all serum samples, the binding to G186V was almost as strong as to WT, whereas binding to L194P was consistently weaker than to WT or G186V. We also tested binding of the HA-RBS-targeted antibody C05 (Ekiert et al., 2012) to Bris07 WT, G186V, and L194P recombinant HA proteins using biolayer interferometry (BLI) (Figure 6B). C05 exhibited high affinity to WT (K_d = 1.0 ± 0.1 nM) and to G186V (K_d = 1.4 ± 0.2 nM), but very weak binding to L194P (>1,000 nM). As a positive control, the stem-binding antibody CR9114 (Dreyfus et al., 2012) bound equally well to all three recombinant proteins (Figure S4B). Overall, these results substantiate the conclusion that G186V, unlike L194P (Chen et al., 2010b; Popova et al., 2012; Wu et al., 2017b), exhibits minimal antigenic change (Barman et al., 2015; Lu et al., 2006, 2005; Parker et al., 2016).

DISCUSSION

Non-additivity of mutational fitness effects is known as epistasis. Our previous studies have shown that epistasis is prevalent in the HA RBS (Wu et al., 2017a) and is relevant to antigenic drift of circulating human H3N2 influenza viruses (Wu et al., 2018). This study further demonstrates that epistasis also exists between egg-adaptive mutations in the HA RBS. Mutational incompatibility between G186V and L194P is an extreme form of epistasis and can be attributed to their opposing structural effects. It is perhaps surprising that human H3N2 virus can adapt to eggs via two mutually exclusive pathways. Egg adaptation of influenza virus involves a receptor tropism switch, as required also in reverse for successful avian-to-human transmission that underlies human influenza pandemics (Shi et al., 2014). However, it remains to be explored whether multiple mutational strategies can be employed by a single influenza strain to switch receptor specificity during avian-to-human transmission.

While the low effectiveness of egg-based, seasonal influenza vaccines has been attributed to HA egg-adaptive mutations that alter the antigenicity (Raymond et al., 2016; Skowronski et al., 2014; Wu et al., 2017b; Zost et al., 2017), some HA egg-adaptive mutations do not affect antigenicity (Barman et al., 2015; Lu et al., 2006, 2005; Parker et al., 2016; Widjaja et al., 2006). Interestingly, our work here revealed that different HA egg-adaptive mutations may not be compatible with each other. Our results raise the possibility that during egg-based seasonal vaccine production, the emergence of an egg-adaptive mutation with undesirable antigenic properties can be prevented by the presence of another egg-adaptive mutation by taking advantage of epistatic interactions.

Our study provides valuable information for the selection of vaccine seed strains with mutations that not only support

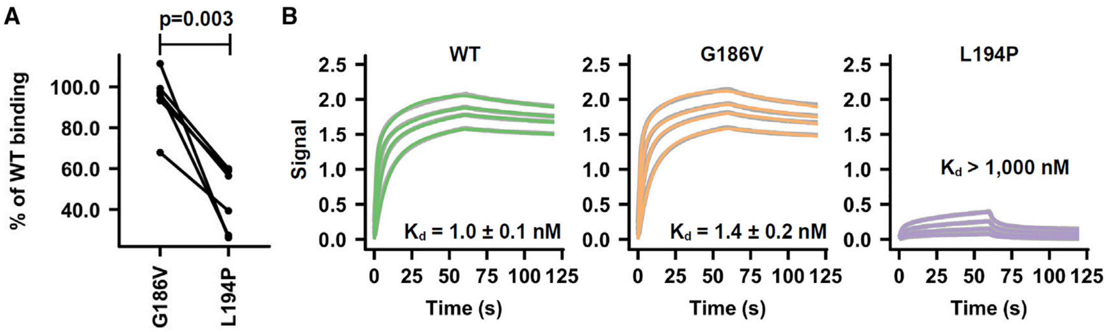

Figure 6. Antigenic Characterization of G186V and L194P

(A) A total of 6 mice were immunized with Bris07 WT virus (6:2 reassortant on a PR8 backbone). Sera from immunized mice were tested for binding to WT, G186V mutant, and L194P mutant of recombinant Bris07 HA using ELISA. Percentage of WT binding was computed as binding level of the mutant at 1:1000 dilution of the serum sample/binding level of WT at 1:1000 dilution of the serum sample. Each line represents one serum sample. Statistical significance was determined using the paired Student's t test.

(B) Biolayer interferometry (BLI) was used to measure the binding kinetics of anti-RBS C05 IgG against recombinant HAs of Bris07 WT, G186V mutant, and L194P mutant. Gray lines represent the response curve and colored lines represent the 1:1 binding model.

efficient replication in eggs but also minimally affect the antigenicity when passaging in eggs. While the ideal approach would be to engineer the desired mutations into the vaccine seed stock using reverse genetics, such an approach is not allowed in certain countries because of their regulation on genetically modified organisms (GMOs). Alternatively, multiple egg-adapted isolates from the same parental strain can be plaque isolated and sequenced. Isolates that carry the desired mutations can then be selected for the vaccine seed strain. Of note, different egg-adapted isolates from the same parental strain often carry different mutations (Barman et al., 2015; Parker et al., 2016). For example, both NYMC X-171 and IVR-147 were derived from the same parental strain Bris07 yet carried different egg-adaptive mutations (Figure 1C). Screening egg-adapted isolates that carry desired mutations may therefore represent a cost-effective approach for selecting the vaccine seed strain. Further studies will need to evaluate the complete inventory of possible egg-adaptive mutations, their antigenicity, and their compatibility with each other. Other related questions that need to be addressed include how different egg-adaptive mutations impact immunogenicity in different age cohorts and how antigenic imprinting (Henry et al., 2019; Lee et al., 2019) influences the immune response elicited by egg-grown influenza vaccines.

Egg-based seasonal influenza vaccines have been the primary preventive against the influenza virus for more than 70 years (Hajj Hussein et al., 2015) despite the caveat of antigenic changes due to egg-adaptive mutations (Raymond et al., 2016; Skowronski et al., 2014; Wu et al., 2017b; Zost et al., 2017). While our work has an immediate impact on the egg-based vaccine seed-strain selection process, a more radical approach is needed in the long run to provide substantial improvements in influenza vaccines. For example, Harding et al. recently developed a dual-HA influenza virus to enable efficient vaccine production in eggs without the need for egg adaptation (Harding et al., 2017). Alternatively, several non-egg-based influenza vaccines have been developed (Harding and Heaton, 2018). Commercialization of cell-based (CDC, 2018a) and recombinant influenza vaccines (CDC, 2018b) that have been developed during the past decade will hopefully completely replace egg-based seasonal vaccines, albeit even if subject to a slow transition. However, the ultimate solution to influenza may require the development of a universal influenza vaccine that can offer long-lasting protection against multiple strains and subtypes (Erbelding et al., 2018).

STAR★METHODS

Detailed methods are provided in the online version of this paper and include the following:

- KEY RESOURCES TABLE
- CONTACT FOR REAGENT AND RESOURCE SHARING
- EXPERIMENTAL MODEL AND SUBJECT DETAILS
  - Cell Cultures
  - Influenza Virus
- METHOD DETAILS
  - Sequence Analysis
  - Construction of Individual Mutants
  - Serial Passages of the Influenza Viruses in Eggs
  - Expression, Crystallization and Structural Determination
  - Glycan Array Analysis
  - Immunization of the Influenza Virus in Mice
  - Antigen Binding Assay
  - IgG Expression and Purification
  - Biolayer Interferometry Binding Assay
- QUANTIFICATION AND STATISTICAL ANALYSIS
- DATA AND SOFTWARE AVAILABILITY

SUPPLEMENTAL INFORMATION

Supplemental Information can be found online at https://doi.org/10.1016/j.chom.2019.04.013.

ACKNOWLEDGMENTS

We thank Steven Head, Jessica Ledesma, and Padmaja Natarajan at TSRI Next Generation Sequencing Core for next-generation sequencing and Henry Tien for technical support with the crystallization robot. We acknowledge NIH R56 AI127371 (I.A.W.), NIH R01 AI114730 (J.C.P.), the Bill and Melinda Gates

Foundation OPP1170236 (I.A.W.), the Bill and Melinda Gates Foundation Post-Doctoral Fellowship in Global Health (N.C.W.), the Theme Based Research Scheme (Ref: T11-705/14N) from Research Grants Council (RGC) of the Hong Kong Special Administrative Region, China, and Guangzhou Medical University High Level University Construction Project Funding (H.L., W.W.S.N., W.L., and C.K.P.M.) for support.

AUTHOR CONTRIBUTIONS

N.C.W., C.K.P.M., and I.A.W. conceived and designed the study. N.C.W. and D.C.W. performed the sequence database analysis. N.C.W. performed the virus rescue experiment. N.C.W., W.W.S.N., and C.K.P.M. performed the egg-passaging experiment. A.J.T., R.M., and J.C.P. performed the glycan array experiment. C.M.N. and J.C.P. synthesized the 3′SLNLN and 6′SLNLN. N.C.W. performed the X-ray crystallography. R.U.K. crystalized the IVR-165 HA. H.L., W.L., and C.K.P.M. performed the mouse immunization and ELISA experiments. N.C.W. performed the biolayer interferometry experiment. N.C.W., C.K.P.M., and I.A.W. wrote the paper, and all authors reviewed and edited the paper.

DECLARATION OF INTERESTS

The authors declare no competing interests.

Received: January 22, 2019
Revised: April 1, 2019
Accepted: April 26, 2019
Published: May 28, 2019

WEB RESOURCES

Global Initiative on Sharing All Influenza Data: https://www.gisaid.org

REFERENCES

Barman, S., Franks, J., Turner, J.C., Yoon, S.W., Webster, R.G., and Webby, R.J. (2015). Egg-adaptive mutations in H3N2v vaccine virus enhance egg-based production without loss of antigenicity or immunogenicity. Vaccine *33*, 3186–3192.

Belongia, E.A., Simpson, M.D., King, J.P., Sundaram, M.E., Kelley, N.S., Osterholm, M.T., and McLean, H.Q. (2016). Variable influenza vaccine effectiveness by subtype: a systematic review and meta-analysis of test-negative design studies. Lancet Infect. Dis. *16*, 942–951.

Broecker, F., Liu, S.T.H., Sun, W., Krammer, F., Simon, V., and Palese, P. (2018). Immunodominance of antigenic site B in the hemagglutinin of the current H3N2 influenza virus in humans and mice. J. Virol. *92*, e01100–01118.

CDC. (2018a). Cell-based flu vaccines. https://www.cdc.gov/flu/protect/vaccine/cell-based.htm.

CDC. (2018b). Recombinant influenza (flu) vaccine. https://www.cdc.gov/flu/protect/vaccine/qa_flublok-vaccine.htm.

Chen, V.B., Arendall, W.B., 3rd, Headd, J.J., Keedy, D.A., Immormino, R.M., Kapral, G.J., Murray, L.W., Richardson, J.S., and Richardson, D.C. (2010a). MolProbity: all-atom structure validation for macromolecular crystallography. Acta Crystallogr. D Biol. Crystallogr. *66*, 12–21.

Chen, Z., Zhou, H., and Jin, H. (2010b). The impact of key amino acid substitutions in the hemagglutinin of influenza A (H3N2) viruses on vaccine production and antibody response. Vaccine *28*, 4079–4085.

Couceiro, J.N., Paulson, J.C., and Baum, L.G. (1993). Influenza virus strains selectively recognize sialyloligosaccharides on human respiratory epithelium; the role of the host cell in selection of hemagglutinin receptor specificity. Virus Res. *29*, 155–165.

Dreyfus, C., Laursen, N.S., Kwaks, T., Zuijdgeest, D., Khayat, R., Ekiert, D.C., Lee, J.H., Metlagel, Z., Bujny, M.V., Jongeneelen, M., et al. (2012). Highly conserved protective epitopes on influenza B viruses. Science *337*, 1343–1348.

Ekiert, D.C., Friesen, R.H., Bhabha, G., Kwaks, T., Jongeneelen, M., Yu, W., Ophorst, C., Cox, F., Korse, H.J., Brandenburg, B., et al. (2011). A highly conserved neutralizing epitope on group 2 influenza A viruses. Science *333*, 843–850.

Ekiert, D.C., Kashyap, A.K., Steel, J., Rubrum, A., Bhabha, G., Khayat, R., Lee, J.H., Dillon, M.A., O'Neil, R.E., Faynboym, A.M., et al. (2012). Cross-neutralization of influenza A viruses mediated by a single antibody loop. Nature *489*, 526–532.

Emsley, P., Lohkamp, B., Scott, W.G., and Cowtan, K. (2010). Features and development of coot. Acta Crystallogr. D Biol. Crystallogr. *66*, 486–501.

Erbelding, E.J., Post, D.J., Stemmy, E.J., Roberts, P.C., Augustine, A.D., Ferguson, S., Paules, C.I., Graham, B.S., and Fauci, A.S. (2018). A universal influenza vaccine: the strategic plan for the National Institute of Allergy and Infectious Diseases. J. Infect. Dis. *218*, 347–354.

Hajj Hussein, I., Chams, N., Chams, S., El Sayegh, S., Badran, R., Raad, M., Gerges-Geagea, A., Leone, A., and Jurjus, A. (2015). Vaccines through centuries: major cornerstones of global health. Front. Public Health *3*, 269.

Harding, A.T., Heaton, B.E., Dumm, R.E., and Heaton, N.S. (2017). Rationally designed influenza virus vaccines that are antigenically stable during growth in eggs. mBio *8*, e00669–00617.

Harding, A.T., and Heaton, N.S. (2018). Efforts to improve the seasonal influenza vaccine. Vaccines (Basel) *6*, E19.

Hartgroves, L.C., Koudstaal, W., McLeod, C., Moncorgé, O., Thompson, C.I., Ellis, J., Bull, C., Havenga, M.J., Goudsmit, J., and Barclay, W.S. (2010). Rapid generation of a well-matched vaccine seed from a modern influenza A virus primary isolate without recourse to eggs. Vaccine *28*, 2973–2979.

Henry, C., Zheng, N.Y., Huang, M., Cabanov, A., Rojas, K.T., Kaur, K., Andrews, S.F., Palm, A.E., Chen, Y.Q., Li, Y., et al. (2019). Influenza virus vaccination elicits poorly adapted B cell responses in elderly individuals. Cell Host Microbe *25*, 357–366.e6.

Jin, H., Zhou, H., Liu, H., Chan, W., Adhikary, L., Mahmood, K., Lee, M.S., and Kemble, G. (2005). Two residues in the hemagglutinin of A/Fujian/411/02-like influenza viruses are responsible for antigenic drift from A/Panama/2007/99. Virology *336*, 113–119.

Katoh, K., and Standley, D.M. (2013). MAFFT multiple sequence alignment software version 7: improvements in performance and usability. Mol. Biol. Evol. *30*, 772–780.

Lee, J., Paparoditis, P., Horton, A.P., Frühwirth, A., McDaniel, J.R., Jung, J., Boutz, D.R., Hussein, D.A., Tanno, Y., Pappas, L., et al. (2019). Persistent antibody clonotypes dominate the serum response to influenza over multiple years and repeated vaccinations. Cell Host Microbe *25*, 367–376.e5.

Lee, P.S., Ohshima, N., Stanfield, R.L., Yu, W., Iba, Y., Okuno, Y., Kurosawa, Y., and Wilson, I.A. (2014). Receptor mimicry by antibody F045-092 facilitates universal binding to the H3 subtype of influenza virus. Nat. Commun. *5*, 3614.

Lin, Y.P., Xiong, X., Wharton, S.A., Martin, S.R., Coombs, P.J., Vachieri, S.G., Christodoulou, E., Walker, P.A., Liu, J., Skehel, J.J., et al. (2012). Evolution of the receptor binding properties of the influenza A(H3N2) hemagglutinin. Proc. Natl. Acad. Sci. USA *109*, 21474–21479.

Lu, B., Zhou, H., Chan, W., Kemble, G., and Jin, H. (2006). Single amino acid substitutions in the hemagglutinin of influenza A/Singapore/21/04 (H3N2) increase virus growth in embryonated chicken eggs. Vaccine *24*, 6691–6693.

Lu, B., Zhou, H., Ye, D., Kemble, G., and Jin, H. (2005). Improvement of influenza A/Fujian/411/02 (H3N2) virus growth in embryonated chicken eggs by balancing the hemagglutinin and neuraminidase activities, using reverse genetics. J. Virol. *79*, 6763–6771.

McCoy, A.J., Grosse-Kunstleve, R.W., Adams, P.D., Winn, M.D., Storoni, L.C., and Read, R.J. (2007). Phaser crystallographic software. J. Appl. Crystallogr. *40*, 658–674.

McWhite, C.D., Meyer, A.G., and Wilke, C.O. (2016). Sequence amplification via cell passaging creates spurious signals of positive adaptation in influenza virus H3N2 hemagglutinin. Virus Evol. *2*, vew026.

Meyer, W.J., Wood, J.M., Major, D., Robertson, J.S., Webster, R.G., and Katz, J.M. (1993). Influence of host cell-mediated variation on the international surveillance of influenza A (H3N2) viruses. Virology *196*, 130–137.

Murshudov, G.N., Skubák, P., Lebedev, A.A., Pannu, N.S., Steiner, R.A., Nicholls, R.A., Winn, M.D., Long, F., and Vagin, A.A. (2011). REFMAC5 for

the refinement of macromolecular crystal structures. Acta Crystallogr. D Biol. Crystallogr. *67*, 355–367.

Neumann, G., Watanabe, T., Ito, H., Watanabe, S., Goto, H., Gao, P., Hughes, M., Perez, D.R., Donis, R., Hoffmann, E., et al. (1999). Generation of influenza A viruses entirely from cloned cDNAs. Proc. Natl. Acad. Sci. USA *96*, 9345–9350.

Otwinowski, Z., and Minor, W. (1997). Processing of x-ray diffraction data collected in oscillation mode. Methods Enzymol. *276*, 307–326.

Parker, L., Wharton, S.A., Martin, S.R., Cross, K., Lin, Y., Liu, Y., Feizi, T., Daniels, R.S., and McCauley, J.W. (2016). Effects of egg-adaptation on receptor-binding and antigenic properties of recent influenza A (H3N2) vaccine viruses. J. Gen. Virol. *97*, 1333–1344.

Peng, W., de Vries, R.P., Grant, O.C., Thompson, A.J., McBride, R., Tsogtbaatar, B., Lee, P.S., Razi, N., Wilson, I.A., Woods, R.J., et al. (2017). Recent H3N2 viruses have evolved specificity for extended, branched human-type receptors, conferring potential for increased avidity. Cell Host Microbe *21*, 23–34.

Popova, L., Smith, K., West, A.H., Wilson, P.C., James, J.A., Thompson, L.F., and Air, G.M. (2012). Immunodominance of antigenic site B over site A of hemagglutinin of recent H3N2 influenza viruses. PLoS One *7*, e41895.

Raymond, D.D., Stewart, S.M., Lee, J., Ferdman, J., Bajic, G., Do, K.T., Ernandes, M.J., Suphaphiphat, P., Settembre, E.C., Dormitzer, P.R., et al. (2016). Influenza immunization elicits antibodies specific for an egg-adapted vaccine strain. Nat. Med. *22*, 1465–1469.

Shi, Y., Wu, Y., Zhang, W., Qi, J., and Gao, G.F. (2014). Enabling the 'host jump': structural determinants of receptor-binding specificity in influenza A viruses. Nat. Rev. Microbiol. *12*, 822–831.

Skowronski, D.M., Janjua, N.Z., De Serres, G., Sabaiduc, S., Eshaghi, A., Dickinson, J.A., Fonseca, K., Winter, A.L., Gubbay, J.B., Krajden, M., et al. (2014). Low 2012-13 influenza vaccine effectiveness associated with mutation in the egg-adapted H3N2 vaccine strain not antigenic drift in circulating viruses. PLoS One *9*, e92153.

Sriwilaijaroen, N., Kondo, S., Yagi, H., Wilairat, P., Hiramatsu, H., Ito, M., Ito, Y., Kato, K., and Suzuki, Y. (2009). Analysis of N-glycans in embryonated chicken egg chorioallantoic and amniotic cells responsible for binding and adaptation of human and avian influenza viruses. Glycoconj. J. *26*, 433–443.

Stevens, J., Chen, L.M., Carney, P.J., Garten, R., Foust, A., Le, J., Pokorny, B.A., Manojkumar, R., Silverman, J., Devis, R., et al. (2010). Receptor specificity of influenza A H3N2 viruses isolated in mammalian cells and embryonated chicken eggs. J. Virol. *84*, 8287–8299.

Widjaja, L., Ilyushina, N., Webster, R.G., and Webby, R.J. (2006). Molecular changes associated with adaptation of human influenza A virus in embryonated chicken eggs. Virology *350*, 137–145.

Wiley, D.C., Wilson, I.A., and Skehel, J.J. (1981). Structural identification of the antibody-binding sites of Hong Kong influenza haemagglutinin and their involvement in antigenic variation. Nature *289*, 373–378.

Wilson, I.A., Skehel, J.J., and Wiley, D.C. (1981). Structure of the haemagglutinin membrane glycoprotein of influenza virus at 3 Å resolution. Nature *289*, 366–373.

Wu, N.C., Thompson, A.J., Xie, J., Lin, C.W., Nycholat, C.M., Zhu, X., Lerner, R.A., Paulson, J.C., and Wilson, I.A. (2018). A complex epistatic network limits the mutational reversibility in the influenza hemagglutinin receptor-binding site. Nat. Commun. *9*, 1264.

Wu, N.C., and Wilson, I.A. (2017). A perspective on the structural and functional constraints for immune evasion: insights from influenza virus. J. Mol. Biol. *429*, 2694–2709.

Wu, N.C., Xie, J., Zheng, T., Nycholat, C.M., Grande, G., Paulson, J.C., Lerner, R.A., and Wilson, I.A. (2017a). Diversity of functionally permissive sequences in the receptor-binding site of influenza hemagglutinin. Cell Host Microbe *21*, 742–753.e8.

Wu, N.C., Zost, S.J., Thompson, A.J., Oyen, D., Nycholat, C.M., McBride, R., Paulson, J.C., Hensley, S.E., and Wilson, I.A. (2017b). A structural explanation for the low effectiveness of the seasonal influenza H3N2 vaccine. PLoS Pathog. *13*, e1006682.

Zost, S.J., Parkhouse, K., Gumina, M.E., Kim, K., Diaz Perez, S., Wilson, P.C., Treanor, J.J., Sant, A.J., Cobey, S., and Hensley, S.E. (2017). Contemporary H3N2 influenza viruses have a glycosylation site that alters binding of antibodies elicited by egg-adapted vaccine strains. Proc. Natl. Acad. Sci. USA *114*, 12578–12583.

STAR★METHODS

KEY RESOURCES TABLE

REAGENT or RESOURCE	SOURCE	IDENTIFIER
DMEM medium	Thermo Fisher Scientific	Cat#11995065
DMEM/F12 medium	Thermo Fisher Scientific	Cat#11320033
ExpiCHO Expression System Kit	Thermo Fisher Scientific	Cat#A29133
HyClone insect cell culture medium	GE Healthcare	Cat#SH30280.03
MEM non-essential amino acids	Thermo Fisher Scientific	Cat#11140050
Trypsin-EDTA	Thermo Fisher Scientific	Cat#25200056
Penicillin-Streptomycin	Thermo Fisher Scientific	Cat#15140122
Fetal Bovine Serum (FBS)	Thermo Fisher Scientific	Cat#16000044
Phosphate-buffered saline (PBS)	Thermo Fisher Scientific	Cat#14040133
Lipofactamine 2000	Thermo Fisher Scientific	Cat#11668019
Ni-NTA Superflow	Qiagen	Cat#30450
HA protein sequences	GISAID; http://gisaid.org	N/A
DH10Bac competent cells	Thermo Fisher Scientific	Cat#10361012
BALB/c mice	Laboratory Animal Unit at HKU	N/A
Antibodies		
C05	(Ekiert et al., 2012)	N/A
CR9114	(Dreyfus et al., 2012)	N/A
Anti-HIS mouse antibody	Thermo Fisher Scientific	Cat#MA1-21315; RRID: AB_557403
Alexa647-linked anti-mouse IgG	Thermo Fisher Scientific	Cat#A-21235; RRID: AB_141693
Chemicals and Recombinant Proteins		
DpnI	New England Biolabs	Cat#R0176L
Trypsin	New England Biolabs	Cat#P8101S
TPCK-Trypsin	Thermo Fisher Scientific	Cat#20233
RNaseOUT	Thermo Fisher Scientific	Cat#10777019
Sodium chloride (NaCl)	Sigma-Aldrich	Cat#S9888
Tris Base	Sigma-Aldrich	Cat#11814273001
Concentrated hydrochloric acid (HCl)	Sigma-Aldrich	Cat#H1758
Sodium azide (NaN_3)	Sigma-Aldrich	Cat#S2002
Bovine Serum Albumin (BSA)	Sigma-Aldrich	Cat#A9418
Tween 20	Fisher Scientific	Cat#BP337-500
3’SLNLN	In-house synthesis	N/A
6’SLNLN	In-house synthesis	N/A
Chemicals for protein crystallization	Hampton Research	N/A
Addavax	InvivoGen	Cat#vac-adx-10
Critical Commercial Assays		
In-Fusion HD Cloning Kit	Takara	Cat#639647
KOD Hot Start DNA Polymerase	EMD Millipore	Cat#71086-3
PCR Clean-Up and Gel Extraction Kit	Clontech Laboratories	Cat#740609.250
QIAprep Spin Miniprep Kit	Qiagen	Cat#27106
NucleoBond Xtra Maxi	Clontech Laboratories	Cat#740414.100
Superscript III reverse transcriptase	Thermo Fisher Scientific	Cat#18080044
QIAamp Viral RNA Mini Kit	Qiagen	Cat#52904
QuikChange XL Mutagenesis kit	Stratagene	Cat#200516

(*Continued on next page*)

Continued

REAGENT or RESOURCE	SOURCE	IDENTIFIER
**Deposited Data**		
Raw sequencing reads	This study	BioProject PRJNA532726
X-ray coordinates and structure factors	This study	PDB: 6NS9, 6NSA, 6NSB, 6NSC, 6NSF, 6NSG
**Cell Lines**		
HEK 293T cells	N/A	N/A
MDCK-SIAT1 cells	Sigma-Aldrich	Cat#05071502-1VL
HEK 293S GnTI$^{-/-}$ cells	ATCC	ATCC CRL-3022
ExpiCHO cells	Thermo Fisher Scientific	Cat#A29127
Sf9 cells	ATCC	ATCC CRL-1711
High Five cells	Thermo Fisher Scientific	Cat#B85502
**Oligonucleotides**		
Bris07-G186V-F: 5'-GGG GTT CAC CAC CCG GTT ACG GAC AAT GAC CAA-3'	Integrated DNA Technologies	N/A
Bris07-G186V-R: 5'-TTG GTC ATT GTC CGT AAC CGG GTG GTG AAC CCC-3'	Integrated DNA Technologies	N/A
Bris07-L194P-F: 5'-AAT GAC CAA ATC TTC CCG TAT GCT CAA GCA TCA-3'	Integrated DNA Technologies	N/A
Bris07-L194P-R: 5'-TGA TGC TTG AGC ATA CGG GAA GAT TTG GTC ATT-3'	Integrated DNA Technologies	N/A
Vic11-G186V-F: 5'-GGG GTT CAC CAC CCG GTT ACG GAC AAG GAC CAA-3'	Integrated DNA Technologies	N/A
Vic11-G186V-R: 5'-TTG GTC CTT GTC CGT AAC CGG GTG GTG AAC CCC-3'	Integrated DNA Technologies	N/A
Vic11-L194P-F: 5′-AAG GAC CAA ATC TTC CCG TAT GCT CAA TCA TCA-3′	Integrated DNA Technologies	N/A
Vic11-L194P-R: 5′-TGA TGA TTG AGC ATA CGG GAA GAT TTG GTC CTT-3′	Integrated DNA Technologies	N/A
**Recombinant DNA**		
pFUSE-CHIg-hG1	InvivoGen	Cat#pfuse-hchg1
pFUSE2-CLIg-hK	InvivoGen	Cat#pfuse2-hclk
WSN 8-plasmid reverse genetics	Neumann et al., 1999	N/A
PR8 8-plasmid reverse genetics	Neumann et al., 1999	N/A
pHW2000-chimeric Bris07 HA	Wu et al., 2018	N/A
pHW2000-chimeric Vic11 HA	Wu et al., 2018	N/A
pFast-Bris07 (H3 HA)	Ekiert et al., 2012	N/A
pFast-Vic11 (H3 HA)	Ekiert et al., 2012	N/A
pFast-IVR-165 (H3 HA)	This study	N/A
**Software and Algorithms**		
R	https://www.r-project.org	RRID: SCR_001905
Python	https://www.python.org	RRID: SCR_008394
MAFFT version 7.157b	Katoh and Standley, 2013	RRID: SCR_011811
HKL2000	Otwinowski and Minor, 1997	RRID: SCR_015547
Phaser	McCoy et al., 2007	RRID: SCR_014219

(*Continued on next page*)

Continued

REAGENT or RESOURCE	SOURCE	IDENTIFIER
Coot	Emsley et al., 2010	RRID: SCR_014222
Refmac5	Murshudov et al., 2011	RRID: SCR_014225
MolProbity	Chen et al., 2010a	RRID: SCR_014226
Custom scripts	This study	https://github.com/wchnicholas/incompatible_egg_muts

CONTACT FOR REAGENT AND RESOURCE SHARING

Further information and requests for resources and reagents should be directed to and will be fulfilled by the Lead Contact, Ian A. Wilson (wilson@scripps.edu).

EXPERIMENTAL MODEL AND SUBJECT DETAILS

Cell Cultures

HEK 293T cells (human embryonic kidney cells, female) were maintained in DMEM medium supplemented with 10% fetal bovine serum (FBS), 1x MEM non-essential amino acids, and 100 U mL^{-1} of Penicillin-Streptomycin. MDCK-SIAT1 cells (Madin-Darby canine hidney cells with stable expression of human 2,6-sialtransferase, female) were maintained in DMEM medium supplemented with 10% FBS, 1x MEM non-essential amino acids, and 100 U mL^{-1} of Penicillin-Streptomycin. Sf9 cells (*Spodoptera frugiperda* ovarian cells, female) and High Five cells (*Trichoplusia ni* ovarian cells, female) were maintained HyClone insect cell culture medium. ExpiCHO cells (Chinese hamster ovary cells, female) were maintained according to the manufacturer's instructions (Thermo Fisher Scientific). HEK 293S GnTI$^{-/-}$ cells (human embryonic kidney cells, female) were maintained in DMEM/F12 medium supplemented with 10% FBS, and 100 U mL^{-1} of Penicillin-Streptomycin.

Influenza Virus

Recombinant influenza virus was generated based on the A/WSN/33 eight-plasmid reverse genetic system (Neumann et al., 1999). In this study, chimeric 6:2 reassortants were employed with the hemagglutinin (HA) and neuraminidase (NA) ectodomains being replaced by those from H3N2 viruses (Wu et al., 2017a). The HA protein sequence of A/Brisbane/10/2007 (Bris07) wild type (WT) was identical to GenBank: ABW23422.1, which did not contain any egg-adaptive mutations. The HA protein sequence of IVR-165 was identical to GISAID: EPI551807. Transfection was performed in HEK 293T/MDCK-SIAT1 cells (Sigma-Aldrich, catalog number: 05071502-1VL) co-culture (ratio of 6:1) at 60% confluence using lipofectamine 2000 (Life Technologies) according to the manufacturer's instructions. At 24 hours post-transfection, cells were washed twice with phosphate-buffered saline (PBS) and cell culture medium was replaced with OPTI-MEM medium supplemented with 0.8 $\mu g\ mL^{-1}$ tosyl phenylalanyl chloromethyl ketone (TPCK)-trypsin. Virus was harvested at 72 h post-transfection. For measuring virus titer by the $TCID_{50}$ (median tissue culture infectious dose) assay, MDCK-SIAT1 cells were washed twice with PBS prior to the addition of virus, and OPTI-MEM medium was supplemented with 0.8 $\mu g\ mL^{-1}$ TPCK-trypsin. The virus used for immunization and egg passaging was generated in the same manner, except that the A/PR/8/34 (PR8) eight-plasmid reverse genetic system was used instead to generate the 6:2 reassortant (Neumann et al., 1999).

METHOD DETAILS

Sequence Analysis

A total of 45,218 full-length human H3N2 HA protein sequences were downloaded from the Global Initiative for Sharing Avian Influenza Data (GISAID; http://gisaid.org). Sequences with ambiguous amino acids were removed. Sequence alignment was performed by MAFFT version 7.157b (Katoh and Standley, 2013). Passaging history was determined by parsing regular expression in FASTA headers as described (McWhite et al., 2016). Egg-adaptive mutations for a given position were defined as amino-acid variants that were observed only in egg-passaged isolates but not in isolates without any passage in a given year. Those egg-adaptive mutations that were observed in 5 out of 16 years (from 2003 to 2018) were classified as major egg-adaptive mutations.

Construction of Individual Mutants

Individual mutants for validation experiments were constructed using the QuikChange XL Mutagenesis kit (Stratagene) according to the manufacturer's instructions.

Serial Passages of the Influenza Viruses in Eggs

For each passaging experiment, three nine- to eleven-day embryonated chicken eggs (specific pathogen free) were inoculated with 0.2 ml of 10^5 plaque-forming unit (PFU) ml^{-1} PR8-A/Brisbane/10/2007 (6:2 reassortant) HA mutant virus at 37°C for 48 hours. The

allantoic fluid from each egg was collected and pooled. Five serial passages were performed for each passaging experiment. The viral RNA was extracted from the allantoic fluid using QIAamp Viral RNA Mini Kit (Qiagen Sciences). The extracted RNA was then reverse transcribed to cDNA using Superscript III reverse transcriptase (Life Technologies). The nucleotide region corresponding to the HA receptor-binding domain was amplified by polymerase chain reaction (PCR) using KOD DNA polymerase (EMD Millipore) according to the manufacturer's instructions, with primers: 5'-CAC TCT TTC CCT ACA CGA CGC TCT TCC GAT CTX XXG GTC ACT AGT TGC CTC ATC CGG-3' and 5'-GAC TGG AGT TCA GAC GTG TGC TCT TCC GAT CTG GTG CAT CTG ATC TCA TTA TTG-3'. The sequence XXX represents the barcode sequence for distinguishing samples from different passages. A second PCR was performed to add the rest of the adapter sequence and index to the amplicon using primers: 5'-AAT GAT ACG GCG ACC ACC GAG ATC TAC ACT CTT TCC CTA CAC GAC GCT-3' and 5'-CAA GCA GAA GAC GGC ATA CGA GAT XXX XXX GTG ACT GGA GTT CAG ACG TGT GCT-3'. Positions annotated by an "X" represented the index sequence for distinguishing different passaging experiments. The final PCR products were submitted for next-generation sequencing using Illumina MiSeq PE300.

Expression, Crystallization and Structural Determination

Briefly, the HA ectodomain, which corresponds to 11–329 (HA1) and 1–176 (HA2) based on H3 numbering, was fused with an N-terminal gp67 signal peptide and a C-terminal BirA biotinylation site, thrombin cleavage site, trimerization domain, and a His_6 tag, and then cloned into a customized baculovirus transfer vector (Ekiert et al., 2011). The HA protein sequence of A/Brisbane/10/2007 (Bris07) wild type (WT) was identical to GenBank: ABW23422.1, which did not contain any egg-adaptive mutations. The HA protein sequence of IVR-165 was identical to GISAID: EPI551807. Bris07 mutants were derived from Bris07 WT using QuikChange XL Mutagenesis kit (Stratagene, see above). Recombinant bacmid DNA was generated using the Bac-to-Bac system (Life Technologies). Baculovirus was generated by transfecting purified bacmid DNA into Sf9 cells using FuGene HD (Promega) (Southampton, UK). HA was expressed by infecting suspension cultures of High Five cells (Life Technologies) with baculovirus at an MOI of 5 to 10 and incubating at 28°C with shaking at 110 rpm for 72 hours. The supernatant was concentrated. HA0 was purified by Ni-NTA and buffer exchanged into 20 mM Tris-HCl pH 8.0 and 150 mM NaCl. For crystallization, HA0 was treated with trypsin (New England Biolabs) to remove the C-terminal tag (BirA biotinylation site, thrombin cleavage site, trimerization domain, and the His_6 tag) and to produce the cleaved mature HA (HA1/HA2). The trypsin-digested HA was then purified by size exclusion chromatography on a Hiload 16/90 Superdex 200 column (GE Healthcare) in 10 mM Tris pH 8.0, 50 mM NaCl, and 0.02% NaN_3. HA crystal screening was carried out using our high-throughput, robotic CrystalMation system (Rigaku) using the sitting drop vapor diffusion method at 4°C and 20°C with each drop consisting of 100 nL protein + 100 nL precipitant. Diffraction-quality crystals for IVR-165 (10 mg/ml) were obtained using 44% 2-methyl-2,4-pentanediol, 0.1 M HEPES pH 7.0 as precipitant at 4°C. Diffraction-quality crystals for Bris07 G186V/L194P (10 mg/ml) were obtained from 0.1 M CAPS pH 10.5 and 29% PEG 400 at 20°C. To generate HA-receptor complexes, crystals were soaked in reservoir solution supplemented with 20 mM of receptor analogs for 2 hours. The resulting crystals were flash cooled, and stored in liquid nitrogen until data collection. Diffraction data were collected at the APS GM/CA-CAT 23ID-B and at the ALS 5.0.3, and then indexed, integrated and scaled using HKL2000 (HKL Research, Charlottesville, VA) (Otwinowski and Minor, 1997). The structure was solved by molecular replacement using Phaser (McCoy et al., 2007) with PDB 4O5N (Lee et al., 2014) or PDB 6AOQ (Wu et al., 2017b) as the molecular replacement model, modeled using Coot (Emsley et al., 2010), and refined using Refmac5 (Murshudov et al., 2011). Ramachandran statistics were calculated using MolProbity (Chen et al., 2010a).

Glycan Array Analysis

Recombinant trimeric HA0 was expressed in HEK 293S GnTI$^{-/-}$ cells, purified and analyzed on a glycan array as previously described (Peng et al., 2017). Briefly, soluble trimeric HA (50 μg mL^{-1}) was pre-complexed with the anti-HIS mouse antibody (Thermo Fisher Scientific) and the Alexa647-linked anti-mouse IgG (Thermo Fisher Scientific) at 4:2:1 molar ratio for 15 minutes on ice in 50 μL PBST. This complex was incubated on the array surface in a humidified chamber for 60 minutes before washing and analysis using an Innoscan 1100AL microarray scanner (Innopsys, Chicago, IL). Fluorescent signal intensity was measured using Mapix (Innopsys) and mean intensity minus mean background of 4 replicate spots was calculated. A complete list of the glycans on the array is presented in Data S1.

Immunization of the Influenza Virus in Mice

Group of six 8-10 weeks old BALB/c mice were inoculated with 1,000 PFU of PR8-Bris07 (6:2 reassortant) wild type virus intranasally. At 16 days post-infection, the mice were further boosted by intraperitoneal injection of 1,000 PFU of same virus mixed with equal volume Addavax (InvivoGen, MF59-like Squalene Adjuvant). The sera of the immunized mice were collected at 24 days post-infection. The serum samples were kept in -20°C for further assay. All animal procedures were carried out under institutionally approved protocols (Approval number: 4884-18) at The University of Hong Kong.

Antigen Binding Assay

A 96-well enzyme-linked immunosorbent assay (ELISA) plate (Nunc MaxiSorp) was first coated overnight with 100 ng per well of purified recombinant Bris07 HA protein in PBS buffer. The plates were then blocked with PBS containing 0.1% Tween 20 and 5% non-fat milk powder at room temperature for 2 hours. Each mouse serum sample was 2-fold serial diluted in PBS buffer and then added into the ELISA plates for 2-hour incubation at 37°C. After extensive washing with PBS containing 0.1% Tween 20, each well in the plate was further incubated with the HRP-sheep anti-mouse second antibody (1:5000, GE Healthcare) for 1 hour at 37°C. Followed by

another extensive washing step, 100 μl mixture of solution A and B (R&D Systems) was added into each well. After 15 minutes incubation, the reaction was stopped by adding 50 μl of 2 M H_2SO_4 solution and analyzed by the Sunrise (Tecan, Grödig, Austria) absorbance microplate reader measured at 450 nm wavelength.

IgG Expression and Purification

The C05 (Ekiert et al., 2012) and CR9114 (Dreyfus et al., 2012) heavy chains and light chains were cloned into pFUSE-CHIg-hG1 and pFUSE2-CLIg-hK respectively. The plasmids were co-transfected into ExpiCHO cells (Thermo Fisher Scientific) at 2:1 ratio (light to heavy) using the Max titer protocol as described by the manufacturer's instructions for the ExpiCHO Expression System (Thermo Fisher Scientific). Full-length IgG proteins were purified from the supernatant using protein G column on AKTAexpress (GE Healthcare).

Biolayer Interferometry Binding Assay

An Octet Red instrument (ForteBio) was employed for the biolayer interferometry binding assay. C05 IgG or CR9114 IgG at a concentration of 50 μg mL^{-1} was loaded onto the anti-human IgG Fc Capture (AHC) Biosensors. Binding kinetics were measured against the indicated HA at 250 nM, 500 nM, 1,000 nM, and 2,000 nM. The data were fit with a 1:1 binding model to estimate the K_d.

QUANTIFICATION AND STATISTICAL ANALYSIS

The p-value reported in Figure 5A was computed by the paired Student's t-test using the R software package.

DATA AND SOFTWARE AVAILABILITY

Raw sequencing data have been submitted to the NIH Short Read Archive under accession number: BioProject PRJNA532726. The X-ray coordinates and structure factors have been deposited in the RCSB Protein Data Bank under accession codes 6NS9, 6NSA, 6NSB, 6NSC, 6NSF, 6NSG. Custom python scripts for analyzing the co-occurrence frequencies of egg-adaptive mutations and the next-generation sequencing data have been deposited to https://github.com/wchnicholas/incompatible_egg_muts.

Cell Host & Microbe, Volume *25*

Supplemental Information

Preventing an Antigenically Disruptive Mutation in Egg-Based H3N2 Seasonal Influenza Vaccines by Mutational Incompatibility

Nicholas C. Wu, Huibin Lv, Andrew J. Thompson, Douglas C. Wu, Wilson W.S. Ng, Rameshwar U. Kadam, Chih-Wei Lin, Corwin M. Nycholat, Ryan McBride, Weiwen Liang, James C. Paulson, Chris K.P. Mok, and Ian A. Wilson

Table S1. Number of HA sequences in the egg-adaptive mutation analysis, Related to Figure 1.

Year	No passaged	Egg-passaged
**2003**	33	10
**2004**	9	8
**2005**	41	3
**2006**	18	6
**2007**	78	26
**2008**	37	17
**2009**	269	33
**2010**	138	33
**2011**	227	34
**2012**	348	68
**2013**	425	47
**2014**	713	64
**2015**	1,490	56
**2016**	1,993	15
**2017**	4,985	57
**2018**	2,334	52

Table S2. H3N2 components of influenza vaccines, Related to Figure 1.

Season	Strain name	Accession	Reference
**2006-2007**	A/Wisconsin/67/2005 (NYMC X-161)	GenBank: ACF41911.1	https://www.ema.europa.eu/documents/assessment-report/intanza-epar-public-assessment-report_en.pdf
**2009-2010**	A/Uruguay/716/2007 (NYMC X-175C)	GenBank: AIE52751.1	http://www.who.int/immunization_standards/vaccine_quality/fluzone_sanofi_pasteur_product_insert.pdf
**2010-2011**	A/Victoria/210/2009 (NYMC X-187)	GenBank: ADO29878.1	https://ec.europa.eu/health/documents/community-register/2010/2010081886928/anx_86928_en.pdf
**2011-2012**			https://ec.europa.eu/health/documents/community-register/2012/20120206116749/anx_116749_en.pdf
**2012-2013**	A/Victoria/361/2011 (IVR-165)	GISAID: EPI551807	http://ec.europa.eu/health/documents/community-register/2012/20120822124238/anx_124238_en.pdf
**2013-2014**	A/Texas/50/2012 (NYMC X-223A)	GenBank: AIE52715.1	https://ec.europa.eu/health/documents/community-register/2013/20130924126650/anx_126650_en.pdf
**2014-2015**			https://ec.europa.eu/health/documents/community-register/2014/20140806129489/anx_129489_en.pdf
**2015-2016**	A/South Australia/55/2014 (IVR-175)	GISAID: EPI696967	https://ec.europa.eu/health/documents/community-register/2015/20150825132942/anx_132942_en.pdf
**2016-2017**	A/Hong Kong/4801/2014 (NYMC X-263B)	GISAID: EPI614414	https://ec.europa.eu/health/documents/community-register/2016/20161103136374/anx_136374_en.pdf
**2017-2018**			https://www.ema.europa.eu/documents/product-information/intanza-epar-product-information_en.pdf
**2018**	A/Singapore/INFIMH-16-0019/2016 (NIB-104)	GISAID: EPI1082230	http://www.medsafe.govt.nz/profs/Datasheet/f/fluquadriinj.pdf
**2018-2019**	A/Singapore/INFIMH-16-0019/2016 (IVR-186)	GISAID: EPI1151840	https://www.vaccineshoppecanada.com/document.cfm?file=fluzone_qiv_e_2018.pdf
**2019**	A/Switzerland/8060/2017 (NIB-112)	GISAID: EPI_ISL_331787	http://origin.who.int/influenza/vaccines/virus/candidates_reagents/summary_a_h3n2_cvv-egg_sh19.pdf

Table S3. X-ray data collection and refinement statistics, Related to Figure 3, Figure 4 and Figure 5.

**Data collection**	IVR-165 Apo	IVR-165 + 3'SLNLN	IVR-165 + 6'SLNLN	Bris07 G186V/L194P Apo	Bris07 G186V/L194 + 3'SLNLN	Bris07 G186V/L194 + 6'SLNLN
Beamline	APS 23ID-B	ALS 5.0.3	APS 23ID-B	SSRL 12-2	SSRL 12-2	SSRL 12-2
Wavelength (Å)	1.0332	0.9765	1.0332	0.9795	0.9795	0.9795
Space group	H32	H32	H32	H32	H32	H32
Unit cell parameters (Å and °)	a=b=100.4, c=382.1	a=b=101.5, c=388.1	a=b=101.1, c=387.2	a=b=100.6, c=384.5	a=b=100.2, c=382.8	a=b=100.3, c=383.6
Resolution (Å)	50.00-1.95 (2.01-1.95)[a]	50.00-1.95 (2.02-1.95)[a]	50.00-1.75 (1.81-1.75)[a]	50.00-2.25 (2.33-2.25)[a]	50.00-2.10 (2.18-2.10)[a]	50.00-2.40 (2.49-2.40)[a]
Unique Reflections	54,947 (4,957)[a]	56,657 (5,556)[a]	76,888 (6,920)[a]	36,162 (3,556)[a]	44,480 (4,871)[a]	29,754 (2,921)[a]
Redundancy	18.1 (17.4)[a]	7.5 (7.5)[a]	37.7 (35.5)[a]	19.0 (17.1)[a]	19.2 (19.9)[a]	19.4 (19.4)[a]
Completeness (%)	100.0 (100.0)[a]	99.7 (99.4)[a]	100.0 (100.0)[a]	99.9 (99.9)[a]	100.0 (100.0)[a]	100.0 (100.0)[a]
$<I/\sigma_I>$	26.0 (2.8)[a]	33.5 (3.8)[a]	39.0 (1.8)[a]	29.7 (2.9)[a]	20.6 (2.7)[a]	25.5 (2.7)[a]
R_{sym}[b]	0.15 (0.90)[a]	0.11 (0.99)[a]	0.13 (1.33)[a]	0.10 (0.89)[a]	0.13 (0.95)[a]	0.10 (0.92)[a]
R_{pim}[b]	0.04 (0.22)[a]	0.04 (0.38)[a]	0.02 (0.23)[a]	0.02 (0.22)[a]	0.03 (0.21)[a]	0.02 (0.21)[a]
$CC_{1/2}$[c]	1.00 (0.92)[a]	1.00 (0.86)[a]	1.00 (0.92)[a]	1.00 (0.92)[a]	1.00 (0.89)[a]	1.00 (0.92)[a]
Z_a[d]	1	1	1	1	1	1
**Refinement statistics**						
Resolution (Å)	41.86-1.95	42.85-1.95	39.90-1.75	46.82-2.25	46.26-2.10	43.14-2.40
Reflections (work)	51,993	53,635	73,049	34,376	41,587	28,205
Reflections (test)	2,670	2,840	3,825	1,743	2,185	1,448
R_{cryst}(%)[e] / R_{free}(%)[f]	16.7 / 19.3	17.9 / 20.8	16.5 / 18.7	19.8 / 22.8	18.2 / 21.4	18.7 / 23.1
No. of atoms						
Protein	3,975	3,955	3,981	3,898	3,944	3,897
Water	406	423	518	240	368	172
Glycan	201	259	215	183	208	197
Ligand[g]						
Sialic acid	-	20	20	-	21	21
Non-sialic acid	-	37	26	-	-	-
Average *B*-value (Å^2)						
Protein	43	39	35	64	50	69
Water	45	46	45	51	47	53
Glycan	77	76	67	101	88	111
Ligand[g]						
Sialic acid	-	66	59	-	104	156
Non-sialic acid	-	109	84	-	-	-
Wilson *B*-value (Å^2)	25	28	25	36	29	41
**RMSD from ideal geometry**						
Bond length (Å)	0.010	0.010	0.011	0.011	0.010	0.010
Bond angle (°)	1.49	1.51	1.53	1.46	1.48	1.48
**Ramachandran statistics (%)**						
Favored	96.2	96.2	96.4	95.5	95.4	95.1
Outliers	0.0	0.2	0.0	0.0	0.0	0.0
**PDB code**	6NS9	6NSA	6NSB	6NSC	6NSF	6NSG

[a] Numbers in parentheses refer to the highest resolution shell.

[b] $R_{sym} = \Sigma_{hkl} \Sigma_i | I_{hkl,i} - <I_{hkl}> | / \Sigma_{hkl} \Sigma_i I_{hkl,i}$ and $R_{pim} = \Sigma_{hkl} (1/(n-1))^{1/2} \Sigma_i | I_{hkl,i} - <I_{hkl}> | / \Sigma_{hkl} \Sigma_i I_{hkl,i}$, where $I_{hkl,i}$ is the scaled intensity of the ith measurement of reflection h, k, l, $<I_{hkl}>$ is the average intensity for that reflection, and *n* is the redundancy.

[c] $CC_{1/2}$ = Pearson correlation coefficient between two random half datasets.

[d] Z_a is the number of HA protomers per crystallographic asymmetric unit.

[e] $R_{cryst} = \Sigma_{hkl} | F_o - F_c | / \Sigma_{hkl} | F_o |$ x 100, where F_o and F_c are the observed and calculated structure factors, respectively.

[f] R_{free} was calculated as for R_{cryst}, but on a test set comprising 5% of the data excluded from refinement.

[g] Ligand indicates 3'SLNLN or 6'SLNLN

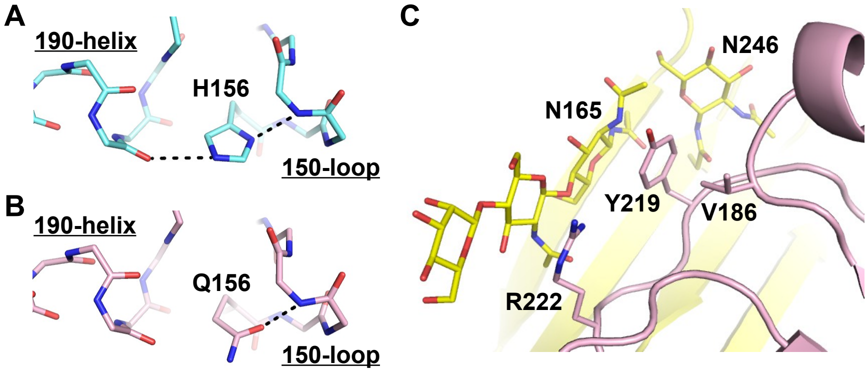

Figure S1. Structural characterization of IVR-165 HA, Related to Figure 3.
(A, B) Hydrogen bond interactions between residue 156 and main chain of the 150-loop (A) in Vic11 HA and in (B) IVR-165 HA are shown. In Vic11 HA, residue 156 also forms a hydrogen bond with the main chain of the 190-helix. Such an interaction is absent in IVR-165 HA.
(C) Stacking interaction between Y219 and the first N-acetyl glucosamine of the N-glycan at residue 165 from the neighboring HA protomer (yellow) is shown. R222 also forms a stacking interaction with the second N-acetyl glucosamine of the same N-glycan at residue 165.

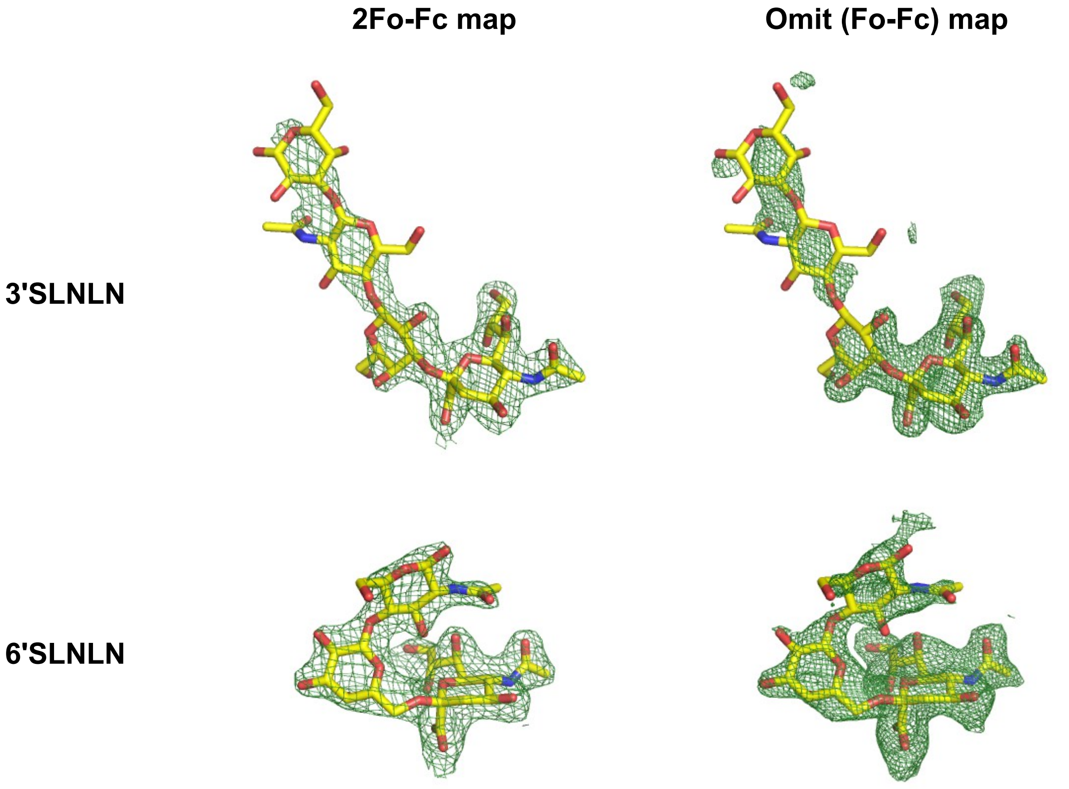

Figure S2. Final 2Fo-Fc and omit (Fo-Fc) electron density maps of glycan receptor analogs (3'SLNLN or 6'SLNLN), Related to Figure 4. Final 2Fo-Fc electron density maps for the glycan receptor analogs (yellow sticks) are represented in a green mesh and contoured at 0.8 σ (left). Omit (Fo-Fc) electron density maps for the glycan receptor analogs are represented in a green mesh and contoured at 2.0 σ (right).

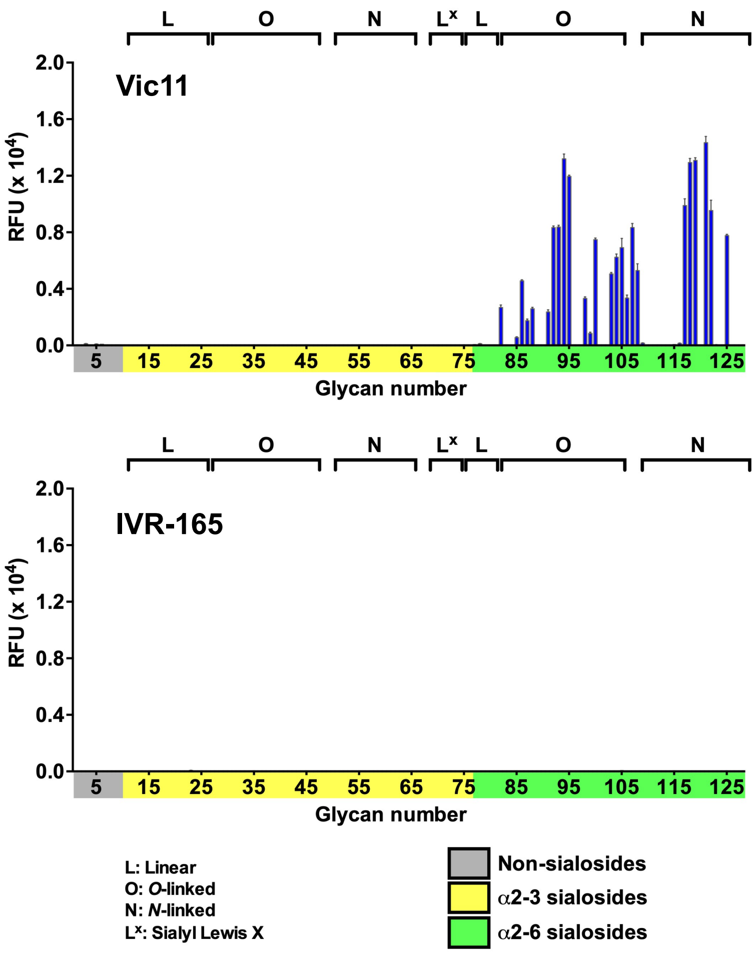

Figure S3. Glycan array analysis of recombinant HA from Vic11 and IVR-165, Related to Figure 4. 293S-expressed recombinant HA was purified and analyzed on the sialoside glycan array.

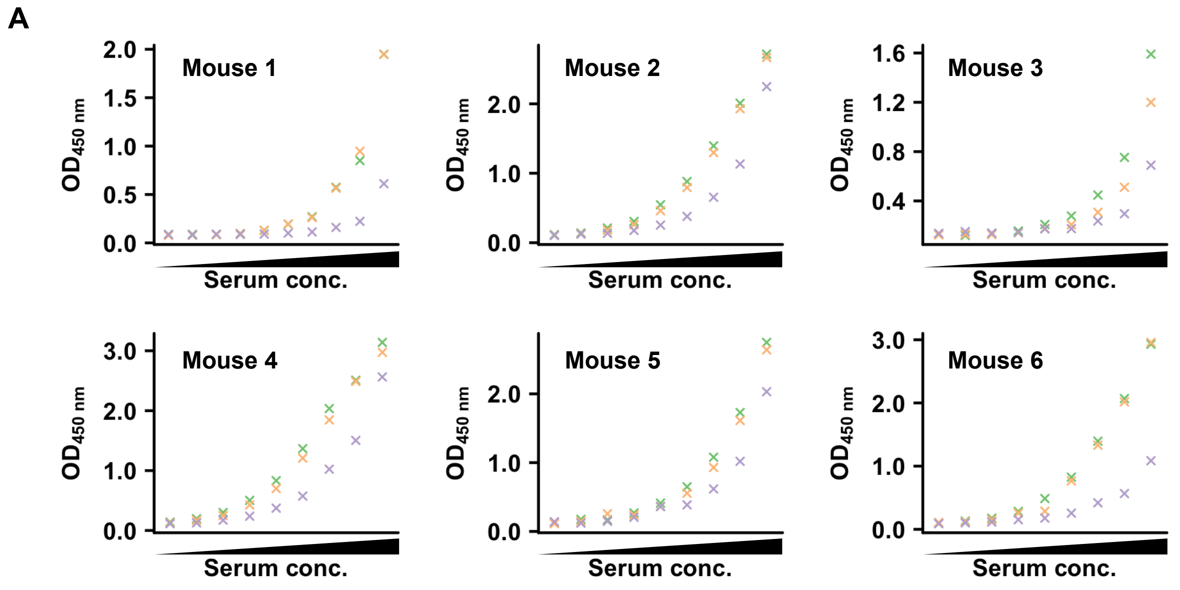

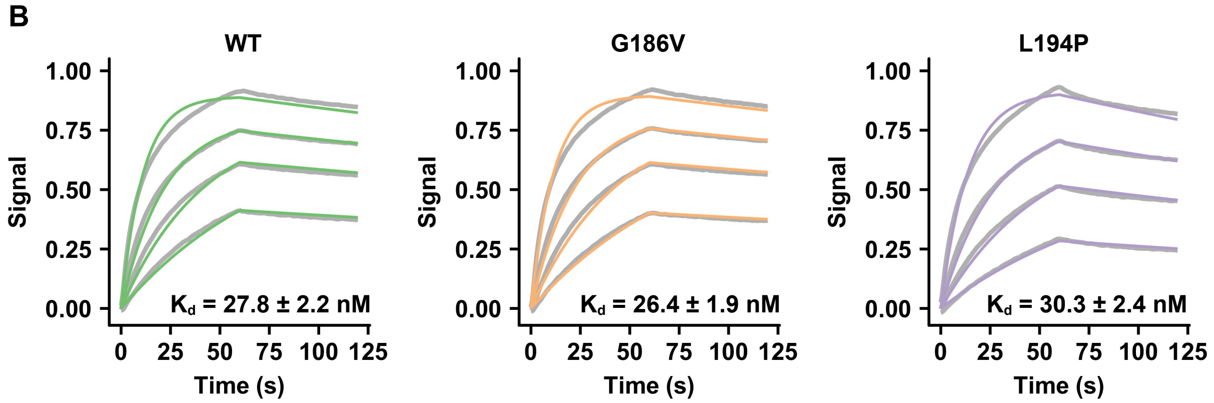

Figure S4. Binding of mouse sera and CR9114 to Bris07 WT or mutant HA, Related to Figure 6.

(A) A total of 6 mice were immunized with Bris07 WT virus (6:2 reassortant on a PR8 backbone). Sera from immunized mice were tested for binding to WT (green), G186V mutant (orange), and L194P mutant (purple) of recombinant Bris07 HA using ELISA. X-axis represents the 2-fold serial dilution of serum at different concentrations (right: highest tested concentration, left: lowest tested concentration). The highest tested concentration was 1:500 dilution of the serum sample (the second highest tested concentration was 1:1000 dilution of the serum sample, and so on).

(B) Biolayer interferometry (BLI) was used to measure the binding kinetics of CR9114 IgG against the recombinant HAs of Bris07 WT, G186V mutant, and L194P mutant. Grey lines represent the response curve and colored lines represent the 1:1 binding model.
